# NAC61 regulates late- and post-ripening osmotic, oxidative, and biotic stress responses in grapevine

**DOI:** 10.1093/jxb/erad507

**Published:** 2023-12-30

**Authors:** Chiara Foresti, Luis Orduña, José Tomás Matus, Elodie Vandelle, Davide Danzi, Oscar Bellon, Giovanni Battista Tornielli, Alessandra Amato, Sara Zenoni

**Affiliations:** Department of Biotechnology, University of Verona, Verona, Italy; Institute for Integrative Systems Biology (I2SysBio), Universitat de València-CSIC, Valencia, Spain; Institute for Integrative Systems Biology (I2SysBio), Universitat de València-CSIC, Valencia, Spain; Department of Biotechnology, University of Verona, Verona, Italy; Department of Biotechnology, University of Verona, Verona, Italy; Department of Biotechnology, University of Verona, Verona, Italy; Department of Biotechnology, University of Verona, Verona, Italy; Department of Biotechnology, University of Verona, Verona, Italy; Department of Biotechnology, University of Verona, Verona, Italy; MPI of Molecular Plant Physiology, Germany

**Keywords:** Abiotic stress, biotic stress, *Botrytis cinerea*, grapevine, late ripening, *NAC61*, post-harvest dehydration, stilbenoid metabolism

## Abstract

During late- and post-ripening stages, grape berry undergoes profound biochemical and physiological changes whose molecular control is poorly understood. Here, we report the role of NAC61, a grapevine NAC transcription factor, in regulating different processes involved in berry ripening progression. NAC61 is highly expressed during post-harvest berry dehydration and its expression pattern is closely related to sugar concentration. The ectopic expression of *NAC61* in *Nicotiana benthamiana* leaves resulted in low stomatal conductance, high leaf temperature, tissue collapse and a higher relative water content. Transcriptome analysis of grapevine leaves transiently overexpressing *NAC61* and DNA affinity purification and sequencing analyses allowed us to narrow down a list of NAC61-regulated genes. Direct regulation of the stilbene synthase regulator *MYB14*, the osmotic stress-related gene *DHN1b*, the *Botrytis cinerea* susceptibility gene *WRKY52*, and *NAC61* itself was validated. We also demonstrate that NAC61 interacts with NAC60, a proposed master regulator of grapevine organ maturation, in the activation of *MYB14* and *NAC61* expression. Overall, our findings establish NAC61 as a key player in a regulatory network that governs stilbenoid metabolism and osmotic, oxidative, and biotic stress responses that are the hallmark of late- and post-ripening grape stages.

## Teaser

NAC61 regulates stilbene biosynthesis and abiotic/biotic stress responses that hallmark late- and post-ripening developmental stages in grapes. NAC61 self-activates and synergistically interacts with the master ripening regulator NAC60.

## Introduction

Fruit ripening is an irreversible, highly regulated process involving physiological and biochemical changes maximizing fruit organoleptic traits to attract herbivores and facilitate seed dispersal (Giovannoni, 2004). The main changes that take place during ripening include fruit degreening, colored pigment accumulation, textural changes (leading to softening), and composition changes, such as the depletion of organic acids and the accumulation of sugars and aroma compounds. This complex program peaks when mature seeds are ready to be dispersed. Nonetheless, at advanced ripening, tissue softening and eventual decay make fruits susceptible to attack by opportunistic pathogens and, consequently, an enhancement of the constitutive defense against pathogens is inherent in the ripening program. Several processes that take place during ripening, such as chloroplast and cell wall disassembly, reactive oxygen species (ROS) increase, protein degradation, and the activation of the secondary metabolism, resemble senescence-like processes (Gómez *et al.*, 2014). However, some specific metabolic activities and the fact that only subsets of senescence-related genes are activated during ripening suggest that, although partially recruiting processes and metabolisms typically associated with senescing tissues, ripening is a distinct process that precedes, and may predispose the fruit to, subsequent senescence (Gapper *et al.*, 2013; Forlani *et al.*, 2019).

To reach its final composition, grape berry, which is a typical non-climacteric fruit, undergoes a developmental process comprising a vegetative and a ripening growth phase (Zenoni *et al.*, 2021). The vegetative phase involves pericarp growth due to rapid cell division and the accumulation of organic acids, tannins, and other phenolic compounds. The ripening phase features several physical, physiological, and compositional changes such as pericarp tissue softening, cell expansion, loss of organic acids, anthocyanin accumulation in the skin, and the progressive accumulation of sugars, reaching levels normally in excess of 20% in the juice (Conde *et al.*, 2007). Several studies have revealed that the onset of ripening, known as veraison, coincides with a profound transcriptomic rearrangement featuring the rapid down-regulation of genes strongly expressed during the vegetative phase of berry development and the up-regulation of genes participating in the ripening program (Fasoli *et al.*, 2012, 2018; Massonnet *et al.*, 2017). Moreover, additional extensive transcriptomic and metabolic changes have been shown to occur in berries of clusters left on the vine beyond ripening, or harvested and placed in dehydrating rooms (Zamboni *et al.*, 2008; Zenoni *et al.*, 2016), thus revealing that the developmental program of the grape berry is not terminated at the fruit commercial ripening stage. These studies identified certain transcription factors (TFs) as putative master regulators of the grape berry developmental progression and the metabolisms featured at each developmental stage (Palumbo *et al.*, 2014; Zenoni *et al.*, 2016; Massonnet *et al.*, 2017; Fasoli *et al.*, 2018). Among these TFs, several members of the NAC (NAM/ATAF1/CUC2) TF gene family are included. NACs are plant-specific TFs with a wide range of activities during plant and fruit development (Olsen *et al.*, 2005; Forlani *et al.*, 2021). The tomato Non-ripening (NOR) was the first NAC TF to be described as a master regulator of fruit ripening (White, 2002; Kumar *et al.*, 2018; Gao *et al.*, 2020), and was recently shown to play a role in leaf senescence as well (Ma *et al.*, 2019). SlNAC1 (also known as SlNAC033) has been shown to have a role in heat and chilling tolerance (Ma *et al.*, 2013; Liang *et al.*, 2015), in defense against bacterial pathogens (Huang *et al.*, 2013), and in fruit softening and pigmentation (Ma *et al.*, 2014; Meng *et al.*, 2016). The Arabidopsis AtNAP (NAC-like, Activated by AP3/PI, ANAC029) has been shown to promote both silique maturation and leaf senescence (Guo and Gan, 2006; Kou *et al.*, 2012). The strawberry FaNAC035 was demonstrated to regulate ripening by controlling fruit softening as well as pigment and sugar accumulation, through the regulation of abscisic acid (ABA) biosynthesis and signalling, and cell-wall degradation and modification (Martín-Pizarro *et al.*, 2021). The NAC TFs are also involved in drought and oxidative stress responses (Tran *et al.*, 2004; Balazadeh *et al.*, 2011; Mao *et al.*, 2012; Puranik *et al.*, 2012) and in the regulation of ROS metabolism (Fang *et al.*, 2015).

In grapevine, the genes *NAC33* and *NAC60* have been functionally investigated as putative master regulators of the vegetative-to-mature transition in several plant organs (D’Incà *et al.*, 2021, 2023). *NAC33* plays a major role in the leaf and fruit, terminating photosynthetic activity and organ growth, whereas for *NAC60*, which is able to complement the *nor* mutant phenotype in tomato, a dual role as an orchestrator of both ripening- and senescence-related processes has been proposed. Moreover, it has been shown that the NAC60 homodimer is the prevalent form in berries during ripening, although the ability of NAC60 to form heterodimers with NAC03 and NAC33 has also been demonstrated, suggesting the existence of a NAC60–NAC regulatory network (D’Incà *et al.*, 2023). Interestingly, *NAC60* and the as yet uncharacterized *NAC61* were also identified as markers of post-harvest dehydration (Zenoni *et al.*, 2016).

Here, we report the functional characterization of the grapevine NAC TF NAC61, providing evidence of its role in the regulation of berry late- and post-ripening processes. We studied the function of NAC61 through investigating the expression and co-expression pattern of *NAC61*, its ectopic overexpression in *Nicotiana benthamiana*, its transient overexpression in grapevine plants, and by performing DNA affinity purification and sequencing (DAP-seq). We identified direct targets of NAC61 and demonstrated its ability to activate genes acting in stilbene biosynthesis and osmotic, heat, and oxidative/biotic stress responses. We also investigated the upstream regulation of *NAC61* and demonstrated its activation by NAC61 itself and by NAC60, showing that abiotic and biotic factors may influence its expression.

## Materials and methods

### Plant material

*Nicotiana benthamiana* plants were grown as previously described (Amato *et al.*, 2019). All the assays and technical measurements were performed on leaves of 5-week-old healthy *N. benthamiana* plants. Different sets of plants were used for each experiment. *Vitis vinifera* cv. ‘Thompson Seedless’ plantlets were micropropagated *in vitro* and cultivated in HB medium (Hoos and Blaich, 1988) in a growth chamber at 25 °C with a 16 h photoperiod. *Vitis vinifera* cv. ‘Thompson Seedless’ embryogenic calli were grown as previously described (Amato *et al.*, 2019). *Vitis vinifera* cv. ‘Syrah’ fruiting cuttings were propagated as previously described (Mullins and Rajasekaran, 1981). *Vitis vinifera* cv. *‘*Corvina’ berries (mature and low-/high-temperature dried) were collected for cDNA preparation and transcriptomic analysis (Shmuleviz *et al.*, 2023). *Vitis vinifera* cv. ‘Müller-Thurgau’ vines were grown in Monzambano (Mantova province, north-east Italy) and grapes were harvested in the 2017 season at full maturity.

### Isolation and cloning

The *NAC61* (*VIT_08s0007g07640*) coding sequence (CDS) was isolated from grapevine cv. ‘Syrah’ ripening berry cDNA, and the regulatory regions of *NAC61*, *DHN1b* (*VIT_04s0023g02480*), *MYB14* (*VIT_07s0005g03340*), and *WRKY52* (*VIT_17s0000g01280*) were isolated from cv. ‘Syrah’ genomic DNA. cDNA and genomic DNA were extracted from cv. ‘Syrah’ fruiting cuttings prepared as previously described (D’Incà *et al.*, 2021). Amplification was performed by using the KAPA HiFi DNA polymerase (KAPA Biosystems, Wilmington, MA, USA) and primer sets listed in Supplementary Table S1. The isolated sequences were directionally cloned into the *pENTR/D-TOPO* Gateway entry vector (Invitrogen, Waltham, MA, USA) and transferred by site-specific LR recombination into a specific binary vector (ThermoFisher Scientific). For agroinfiltration of *N. benthamiana* and grapevine cv. ‘Thompson Seedless’ plantlets, the *NAC61* CDS was transferred into the *pK7GW2.0* binary overexpression vector. For the dual-luciferase reporter assay (DLRA), the *NAC61*, *DHN1b*, *MYB14*, and *WRKY52* target gene regulatory regions were transferred into the *pPGWL7.0* reporter vector to control the expression of the firefly luciferase gene (*LUC*). For the bimolecular fluorescence complementation (BiFC) assay, the *NAC61* sequence was transferred into the *pnYGW* vector.

The *NAC60* CDS isolation, cloning into the *pENTR/D-TOPO* entry vector, and site-specific LR recombination into the *pK7GW2.0* binary overexpression vector was previously performed (D’Incà *et al.* 2023).

### Transient overexpression

The *pK7WG2.0* vectors containing *35S:NAC61* or a non-coding sequence (control) were transferred to *Agrobacterium tumefaciens* strain C58C1 by electroporation. For transient expression in grapevine cv. ‘Thompson Seedless’, 5-week-old *in vitro*-grown plantlets were vacuum infiltrated as previously described (Amato *et al.*, 2016), and molecular analyses were carried out on leaf samples collected 7 d after agroinfiltration. For transient expression in *N. benthamiana*, three fully expanded young leaves were syringe infiltrated as previously described (Amato *et al.*, 2016), and phenotypic analysis was carried out over 3 d after agroinfiltration. To validate the expression of *NAC61*, *N. benthamiana* leaf tissues were collected 2 d after infiltration and immediately pulverized under liquid nitrogen. RNA was isolated from 100 mg of ground leaf material by using TRI Reagent® (Merck) as recommended by the manufacturer. cDNA synthesis was conducted according to Amato *et al.* (2016), and gene expression in comparison to the *ACTIN* internal control was determined by reverse transcription–PCR using the primer sets listed in Supplementary Table S1.

### Stomatal conductance and thermal camera measurements

The stomatal conductance measurements were carried out on control and *35S:NAC61*-expressing *N. benthamiana* leaves for 6 d post-infiltration by using a portable leaf porometer (SC-1, METER Group, Inc., Pullman, WA, USA). Three biological replicates (different plants), each with three technical replicates (different leaves), were performed for each sample (nine replicates for each sample). Thermal images were taken of control and *35S:NAC61*-expressing *N. benthamiana* leaves for 6 d post-infiltration with a thermal camera (FLIR E6 Wifi, FLIR Systems, Sweden). Six biological replicates (different plants), each with three technical replicates (different leaves), were performed for each sample (18 replicates for each sample).

### Relative water content measurement

Relative water content (RWC) measurement was performed on control and *35S:NAC61*-expressing *N. benthamiana* leaves as previously described (Xu *et al.*, 2022). Three biological replicates (different plants), each with three technical replicates (different leaves), were performed for each sample (nine replicates for each sample). Leaves were sampled 2 d after agroinfiltration, immediately weighed (fresh weight; ‘fw’) and then immersed in distilled water for 2 h at room temperature. The weight of hydrated leaves (‘w’) was measured. The leaves were then dried for 24 h at 60 °C and dry weight (‘dw’) was measured. The RWC was calculated as [(fw–dw)/(w–dw)]×100. The evaluation of local water accumulation was performed by the agroinfiltration of *35S:NAC61* and control vectors into delimited portions of the same leaf. Three biological replicates (different plants), each with three technical replicates (different leaves), were performed, resulting in nine replicates for each sample. Infiltrated leaves were inspected daily and photographs of the abaxial face were taken to observe the progression of the phenotype in agroinfiltrated tissues.

### Ion leakage assay

Ion leakage assays were performed on control and *35S:NAC61*-expressing *N. benthamiana* leaf discs according to Imanifard *et al.* (2018). Six biological replicates (different plants), each with three technical replicates (different leaves), were performed for each sample (18 replicates for each sample). Leaf discs (~5 mm in diameter) were collected 24 h after leaf agroinfiltration (T0) and immersed in 50 ml of non-ionic double-distilled water for 30 min at 25 °C with shaking at 90 rpm to eliminate ions released because of physical damage. The 18 leaf discs from each sample were distributed into three wells of a multi-well plate containing 2 ml of distilled water per well (one replicate per well; the six discs were each from an independent plant to avoid plant-specific effects). The conductivity of the solution was measured using a conductivity meter (Horiba Scientific, Edison, NJ, USA) immediately after plate preparation and during the subsequent 24 h under constant light (50 μmol m^–2^ s^–1^) at 25 °C with shaking at 90 rpm.

### 3,3ʹ-Diaminobenzidine assay

3,3ʹ-Diaminobenzidine (DAB) staining was performed on control and *35S:NAC61*-expressing *N. benthamiana* leaves as previously described (Daudi and O’Brien, 2012). Three biological replicates (different plants), each with three technical replicates (different leaves), were performed for each sample, (nine replicates per sample). Leaf discs (~2 cm in diameter) were collected and used for the assay. A DAB solution was used to evaluate the production of H_2_O_2_ 2 d after agroinfiltration. The assay was performed in 12-well plates and the DAB staining solution was vacuum infiltrated. The plate was covered with aluminum foil and incubated for 5 h at room temperature with shaking at 100 rpm. At the end of the staining, the discs were placed in falcon tubes containing 25 ml of 80% ethanol to degrade all the chlorophylls. Finally, the staining was quantified by using ImageJ software (https://imagej.net/ij/index.html).

### Real-time quantitative polymerase chain reaction

Leaf and berry tissues were harvested and pulverized under liquid nitrogen. For gene expression analysis, RNA was isolated from 100 mg of ground leaf material (for the cv. ‘Thompson Seedless’ transcriptomic analysis) and 200 mg of ground berry material (for the expression analysis on cv. ‘Müller-Thurgau’ drying berries), using the Spectrum Plant Total RNA kit (Merck KGaA, Darmstadt, Germany). Gene expression was determined by real-time quantitative polymerase chain reaction (RT–qPCR) as previously described (Zenoni *et al.*, 2011) using the primer sets listed in Supplementary Table S1. Data are presented as the mean ±SD of three biological replicates. Figures show the normalized expression pattern using *UBIQUITIN1* (*UBI1*; *VIT_16s0098g01190*) as internal control. *UBIQUITIN1* has been previously demonstrated to be a good housekeeping gene, while very similar results were obtained with *ELONGATION FACTOR1* (*EF1*; *VIT_12s0035g01130*) in all the experimental conditions. High correlation coefficients (*R*^2^) were obtained by performing linear regression between the *UBI1*- and *EF1*-normalized *NAC61* expression data.

### Transcriptomic analysis on *NAC61* transiently overexpressing grapevine plants

Microarray analysis was performed with the RNA used for RT–qPCR. For transient expression, the three most highly overexpressing plants and three control lines were selected and used as biological replicates. The cDNA synthesis, labelling, hybridization, and washing steps were performed according to the Agilent Microarray-Based Gene Expression Analysis Guide (https://www.agilent.com/cs/library/usermanuals/Public/G4140-90040_GeneExpression_OneColor_6.9.pdf). Each sample was hybridized to an Agilent custom microarray four-pack 44K format (Agilent Sure Print HD 4X44K 60-mer; cat. no. G2514F-048771) (Dal Santo *et al.*, 2016) and scanned using an Agilent Scanner (G2565CA; Agilent Technologies, Santa Clara, CA, USA).

### DNA affinity purification and sequencing

Young leaves of cv. ‘Syrah’ were harvested and pulverized using liquid nitrogen. Genomic DNA was extracted from 1 g of powdered material as described by D’Incà *et al.*, (2021) and Illumina libraries were prepared as previously described (Galli *et al.*, 2018). The *NAC61* sequence was transferred from the *pENTR/D-TOPO* vector to the Gateway-compatible destination vector *pIX-HALO* (Bartlett *et al*., 2017). The HALO-NAC61 and GST-HALO (used as negative control) fusion proteins were translated *in vitro* using the TNT^R^ SP6 coupled reticulocyte lysate system (Promega). Two replicates were used for the TF and input libraries (generated with the GST-HALO empty vector). The DAP-seq was performed according to a previously described procedure (Galli *et al.*, 2018), and a total of 7.0 (control replicate 1), 5.6 (control replicate 2), 24 (NAC61 replicate 1), and 22 (NAC61 replicate 2) million reads were obtained. DAP-seq bioinformatic analysis was performed as previously described (Orduña *et al.*, 2022, 2023; D’Incà *et al.*, 2023). Briefly, DAP-seq libraries were aligned to the PN40024 12X.v2 reference genome using bowtie2 (Langmead and Salzberg, 2012), with post-processing to remove reads with a MAPQ score <30. Peak detection was performed using GEM peak caller (Guo *et al.*, 2012) version 3.4 with the 12X.v2 genome assembly using the following parameters: ‘–q 1 –t 1 –k_min 6 –kmax 20 –k seqs 600 –k_neg_dinu_shuffle’, limited to nuclear chromosomes. The replicates were analyzed as multi-replicates with the GEM replicate mode. Detected peaks were associated to the closest gene model from PN40024 v1 on the 12X.0 assembly transposed to the 12X.2 assembly annotation file using the BioConductor package ChIPpeakAnno (L.J. Zhu *et al.*, 2010) with default parameters.

### Dual-luciferase reporter assay

The *pK7WG2.0* vectors containing the *NAC61* and *NAC60* (*VIT_08s0007g07670*) CDS and the *pPGWL7.0* vectors harboring the *DHN1b*, *MYB14* and *WRKY52* regulative regions were transferred to *A. tumefaciens* strain C58C1 by electroporation. The DLRA was performed on three fully expanded infiltrated *N. benthamiana* leaves from three different plants, as previously described (Cavallini *et al.*, 2015). The assay was performed on fresh leaf discs collected 72 h after *Agrobacterium*-mediated infection and following the manufacturer’s instructions (Promega). A reference vector overexpressing the *Renilla reniformis* luciferase gene (*REN*) was used to normalize LUC luminescence. REN and LUC luminescence were detected using a Tecan Infinite ® M200 PLEX instrument. Each test was performed in biological triplicate and each value was measured in triplicate.

### Grapevine protoplast transfection and bimolecular fluorescence complementation assay

For the BiFC assay, the *NAC61* and *NAC60* CDSs were cloned into the *pnYGW* and *pGWcY* Gateway vectors, respectively. *Vitis vinifera* cv. ‘Thompson Seedless’ protoplasts were isolated from embryogenic calli and transfected (Bertini *et al.*, 2019), cultured in multi-well plates in the dark at 25 °C, and analyzed 1 d after transfection. The yellow fluorescent protein (YFP) signal was detected using a Leica TCS SP5 AOBS confocal microscope (argon laser, 514 nm excitation source, 550–570 nm collection bandwidth, auto gain).

### *Vitis vinifera* cv. ‘Müller-Thurgau’ berry post-harvest dehydration and noble rot induction

Approximately 405 kg of berry bunches of *V. vinifera* cv. ‘Müller-Thurgau’ were harvested in August 2017 in Custoza (Italy) when the total soluble solids content, measured using a DBR35 digital refractometer (Giorgio Bormac, Carpi, Italy), was 18.25 ± 0.05 °Brix. The bunches were arranged in plastic boxes and transferred to a ventilated dehydration facility at the farm ‘La Prendina’ in Monzambano (Mantova, Italy) for dehydration under controlled conditions [14–15 °C, 53–60% relative humidity (RH)] provided by a DEUM 5 HP machine (Sordato, Verona, Italy). Three randomly selected replicates of ~100 berries each were sampled weekly from the start of the trial until 30 d after the induction of noble rot to determine the total soluble solids and the total acidity. In addition, three dedicated boxes were weighed weekly using a CH50K50 electronic balance (Kern, Balingen, Germany). After 29 d of dehydration, half of the boxes were covered with plastic film and water-filled trays were placed inside to increase the RH and induce noble rot (Negri *et al.*, 2017), while the remaining plastic boxes (control berries) were left under normal dehydrating conditions. The two different environmental conditions were imposed for a further 28 d. The RH in both conditions was monitored using Hobo Pro v2 sensors connected to data loggers (Onset Computer Corporation, Bourne, MA, USA). Control and noble-rot-induced berries were sampled in three biological triplicates for transcriptomic analysis at 7, 21, and 28 d (t1, t2, and t3, respectively) from noble rot induction. Each replicate consisted of 50 randomly collected berries that were immediately deseeded and frozen and then pulverized under liquid nitrogen. For measurement of the ratio of glycerol to d-gluconic acid, a glycerol assay kit (Merck) and a d-gluconic acid/d-glucono-δ-lactone assay kit (Megazyme International) were used according to the manufacturers’ instructions. A 1 g sample of powdered berry pericarp material was diluted in 10 ml of buffer containing 500 µl Carrez 1 solution, 500 µl Carrez 2 solution, and 1 ml 0.1 M NaOH topped up to 10 ml with water, and filtered with standard filter paper. After treatment with polyvinylpolypyrrolidone to remove colored solutes, samples were filtered again.

### Gene co-expression networks, binding motif comparison, and promoter analyses

*NAC61* gene co-expression networks (GCNs) were extracted from the AggGCNs app (Orduña *et al.*, 2023). Gene set enrichment analysis conducted in this study was conducted with the gprofiler2 R package (Kolberg *et al.*, 2020), using the MapMan manually curated annotation described by Orduña *et al.* (2023) with default settings. A significance threshold of 0.05 was chosen for *P*-values adjusted with the Benjamini–Hochberg correction procedure (Benjamini and Hochberg, 1995). Enriched sequences found in NAC61 binding sites were compared with those found in *Arabidopsis thaliana* using the RSAT Plants NGS- ChIP-seq Peak-Discovery software (https://rsat.eead.csic.es/plants/) (Santana-Garcia *et al.*, 2022). The top 600 best-scored peak sequences (–50 bp<peak center<+50 bp) were retrieved from the DAP-seq analysis and used with default parameters. Most significant, frequent, and middle-centered motifs were selected. An untargeted binding discovery analysis and a targeted binding comparison analysis were also performed on the *NAC61* promoter using the RSAT Plants Motif discovery oligo-analysis software (https://rsat.eead.csic.es/plants/oligo-analysis_form.cgi), using the default parameters (selecting ‘*Vitis vinifera* PN40024.v4.55’ as organism), and the RSAT Plants Pattern Matching Matrix-scan (https://rsat.eead.csic.es/plants/matrix-scan_form.cgi), for surveying the ‘Arabidopsis PBM’, ‘Athamap’ and ‘Cistrome’ dataset.

### Statistical analysis

We performed pairwise *t*-tests to compare differences in leaf stomatal conductance, thermal imaging, RWC, ion leakage and DAB measurements. To identify differences in gene expression in RT–qPCR and DLRA data we performed one-sample *t*-tests. Feature extraction and statistical analysis of the microarray data were conducted by using the Limma package in R (Ritchie *et al.*, 2015). *P*-values were normalized using the Benjamini–Hochberg correction (Benjamini and Hochberg, 1995) and the differentially expressed genes (DEGs) were identified by adjusted *P*-value <0.1 and selected by fold change (FC) >|1.5|.

## Results

### *NAC61* is up-regulated in post-veraison stages and correlates with osmotic stress in grape berries

**Fig. 1.**
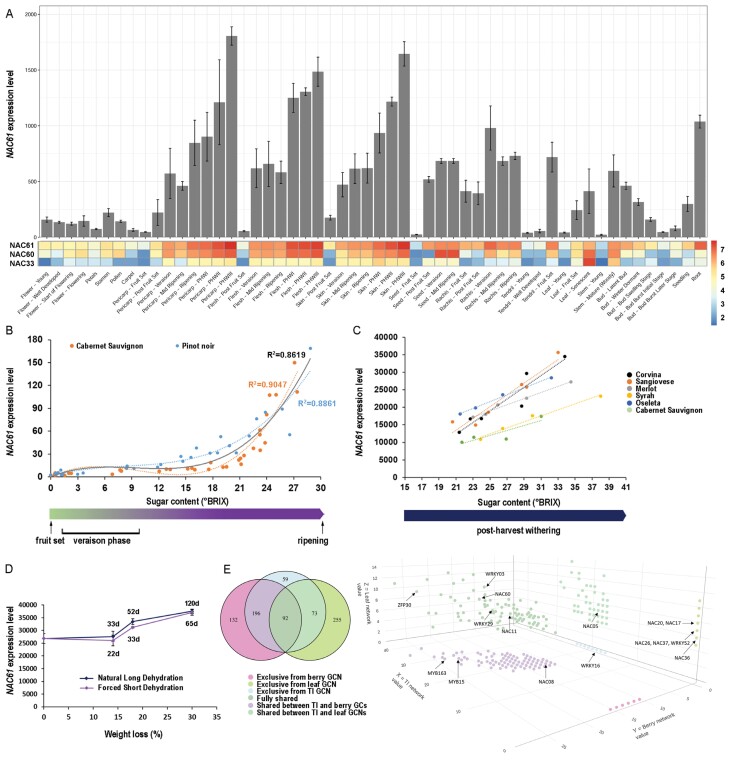
*NAC61* expression analysis. (A) *NAC61* expression behavior in grapevine organs throughout development (bar plot) and compared in the heatmap (logarithmic value) with that of *NAC60* and *NAC33.* The data were retrieved from the atlas transcriptomic dataset of cv. ‘Corvina’ (Fasoli *et al*., 2012). Each value represents the mean ±SD of three biological replicates. (B) Correlation between *NAC61* expression level and sugar content in grape berries sampled from fruit set to maturity in cv. ‘Cabernet Sauvignon’ and cv. ‘Pinot noir’ (Fasoli *et al*., 2018). The black line represents the trend of the averaged values of the two varieties. The R^2^ values shown correspond to the fitting of different polynomial regressions to each corresponding group of samples (orange for cv. ‘Cabernet Sauvignon’ samples, blue for cv. ‘Pinot noir’ samples, and black for the entire set of samples). (C) Correlation between *NAC61* expression level and sugar content in grape berries sampled during post-harvest dehydration in six different varieties (Zenoni *et al*., 2016). (D) Correlation between *NAC61* expression level and berry weight loss in cv. ‘Corvina’ berries sampled during traditional long and forced short post-harvest dehydration processes (Zenoni *et al*., 2020). Expression values were determined by microarray analysis and each value represents the mean ±SD from three biological replicates. (E) *NAC61* GCNs based on berry, leaf, and tissue-independent (TI) datasets. Left, Venn diagram showing exclusive and shared genes based on the three datasets; right, three-dimensional plot of co-expressed genes in which NAC, WRKY, and ZIP family members already described as having involvement in berry ripening and/or stress responses are indicated.

The *NAC61* expression pattern, according to the global gene expression atlas of *V. vinifera* (Fasoli *et al.*, 2012), shows an increase during berry development and also in other organs, such as seeds, rachis, stems and roots, whereas weak expression is found in flower organs (Fig. 1A). In berry tissues of different grape cultivars, the *NAC61* expression level sharply increases at veraison (Fig. 1A; Supplementary Fig. S1A, B) (Massonnet *et al.*, 2017; Fasoli *et al.*, 2018) and shows a second step of up-regulation after harvest, reaching the highest expression level at the end of the post-harvest dehydration process [post-harvest withering (PHW) stages; Fig. 1A]. By examining a transcriptomic dataset from a genotype × environment study (Dal Santo *et al.*, 2018), we observed that *NAC61* belongs to a stage-specific cluster of genes, whose expression increases after veraison and is thus poorly affected by environmental conditions (Supplementary Fig. S1C). We then investigated the relationship between the *NAC61* expression level and the sugar content in berries, based on transcriptomic and technological data retrieved from previous studies. We found a high positive correlation during ripening when the berry exceeds a sugar content of 15–18 °Brix (Fig. 1B) (Fasoli *et al.*, 2018). This close relationship between sugar concentration and *NAC61* expression is maintained until the end of ripening and is also observed in berries during PHW, in which the highest expression level coincides with the highest sugar concentration, independent of the genotype considered (Fig. 1C) (Zenoni *et al.*, 2016). Inspection of the transcriptomic dataset from a study aiming specifically at dissecting the effect of time and dehydration level in post-harvest dehydrating grape berries (Zenoni *et al.*, 2020) revealed that *NAC61* expression is more strongly correlated with berry weight loss level (an indirect measure of sugar concentration) than with time, further strengthening the above-reported observations (Fig. 1D; Supplementary Fig. S1D).

Extracting the *NAC61* gene-centered networks from berry (67 experiments), leaf (42 experiments), and tissue-independent (131 experiments) datasets through the AggGCN app within the VitViz platform (http://www.vitviz.tomsbiolab.com/), we identified a total of 810 *NAC61* co-expressed genes mainly belonging to the ‘Transcription regulation’ and ‘Transcription factors’ functional categories (Supplementary Dataset S1; Supplementary Fig. S2). Most of the NAC61 co-expressed TFs belong to the NAC, zinc finger, WRKY and MYB families (Fig. 1E; Supplementary Dataset S1). Moreover, we found 98 genes previously defined as key regulators of berry ripening and 575 genes (71% of the total) that are differentially modulated during the post-harvest dehydration process (Palumbo *et al.*, 2014; Zenoni *et al.*, 2016; Massonnet *et al.*, 2017; Fasoli *et al.*, 2018) (Supplementary Dataset S1). Accordingly, the stilbene synthases (STSs) regulators *MYB15* (*VIT_05s0049g01020*), *WRKY03* (*VIT_01s0010g03930*), and *WRKY43* (*VIT_14s0068g01770*) are also co-expressed with *NAC61*.

The NAC family multispecies phylogenetic tree (https://tomsbiolab.com/wp-content/uploads/2021/10/Fig.-S4.png) (Supplementary Fig. S3) revealed that *NAC61* is located close to *ANAC046* and *NAC33* (*VIT_19s0027g00230*), both of which are involved in the senescence process (Oda-Yamamizo *et al.*, 2016; D’Incà *et al.*, 2021), to *ORS1*, which codes for an H_2_O_2_-responsive NAC TF also controlling senescence in Arabidopsis (Balazadeh *et al.*, 2011), and to *OsNAC2*, a positive regulator of drought and salt tolerance through ABA-mediated pathways in rice (Jiang *et al.*, 2019).

### *NAC61* heterologous expression induces a water-soaking-like phenotype and programmed cell death in *N. benthamiana* leaves

**Fig. 2.**
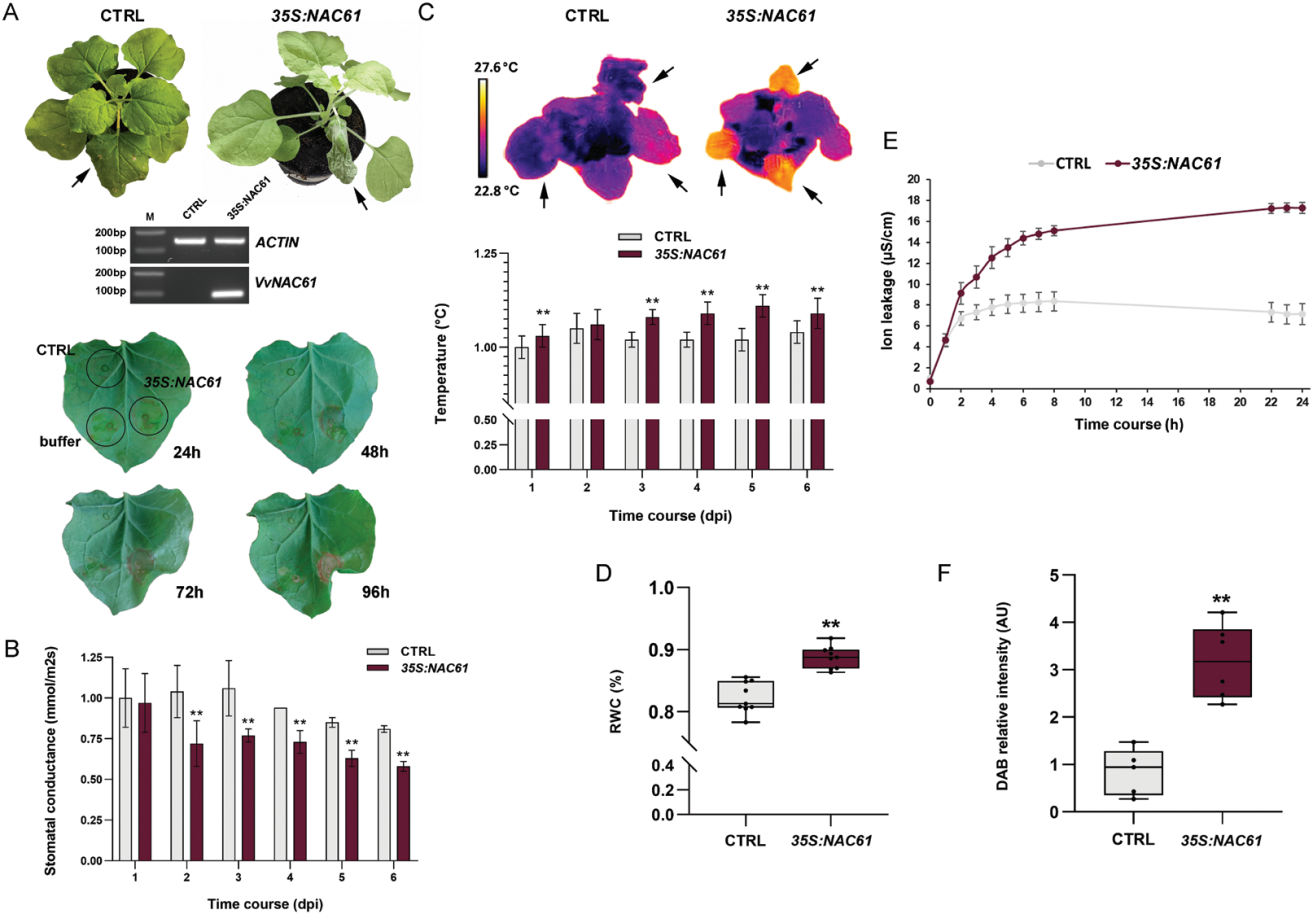
*NAC61* ectopic expression in *N. benthamiana* plants. (A) Control and *NAC61*-expressing *N. benthamiana* plants 3 d after infection (top panel), RT–PCR validating the *NAC61* ectopic expression in comparison to the control (middle panel), and spot-infiltrated leaves after 24, 48, 72, and 96 h (bottom panel). (B) Stomatal conductance measurements in *NAC61*-expressing leaves compared with control leaves. (C) Thermal camera visualization (top panel) and leaf temperature measurements (bottom panel) in *NAC61*-expressing leaves compared with controls. (D) RWC measurements in *NAC61*-expressing leaves compared with controls at 2 d after agroinfiltration. (E) Ion leakage measurements in *NAC61*-expressing leaves compared with controls from T0 (24 h after leaf agroinfiltration) to 24 h. (F) DAB staining determining H_2_O_2_ accumulation in *NAC61*-expressing leaves compared with control leaves at 2 d after agroinfiltration. Each value represents the mean ±SD of three biological replicates tested in technical replicate (*n=*3). Asterisks indicate statistically significant differences (**P<0.01; *t-*test). Data shown in B, C, and E have been normalized to the control value at the starting time point.

The role of *NAC61* was studied through transient heterologous expression in *N. benthamiana* plants. At 3 d after agroinfiltration, transgenic leaves showed leaf tissue collapse with loss of turgor (Fig. 2A). To highlight the effect of NAC61, leaves were simultaneously agroinfiltrated with *NAC61,* the control vector, and the agroinfiltration buffer. At 2 d post-infiltration (48 h), part of the leaves near the site of *NAC61* infiltration showed a darker color, suggesting a local accumulation of water that preceded tissue collapse, observed at 3 d (72 h) and 4 d (96 h) after infiltration (Fig. 2A).

Interestingly, starting from 2 d after *NAC61* agroinfiltration, transgenic leaves displayed a lower stomatal conductance compared with the control leaves (Fig. 2B). In addition, a significantly higher leaf temperature was registered in transgenic leaves (Fig. 2C). To investigate whether *NAC61* expression favors water retention within plant tissue, we measured the RWC in transgenic and control leaves at 2 d after infiltration. The analysis revealed a significantly higher water content in NAC61-overexpressing leaves compared with controls (Fig. 2D), resembling a water-soaking phenotype.

Moreover, in line with the cell death observed at the whole-leaf level at 4 d post-infection (Fig. 2A), *NAC61* expression significantly increased ion leakage as early as 2 d post-infection, suggesting a possible loss of membrane integrity (Fig. 2E). Finally, a higher level of H_2_O_2_ (detected by DAB staining) was observed in *NAC61*-expressing plants in comparison to the controls (Fig. 2F), in line with the above-described phenotype, as H_2_O_2_ is a key ROS involved in both stomatal closure and programmed cell death (Liu and Zhang, 2021).

### Transient *NAC61* overexpression in *V. vinifera* affects stilbenoid-related gene expression

**Fig. 3.**
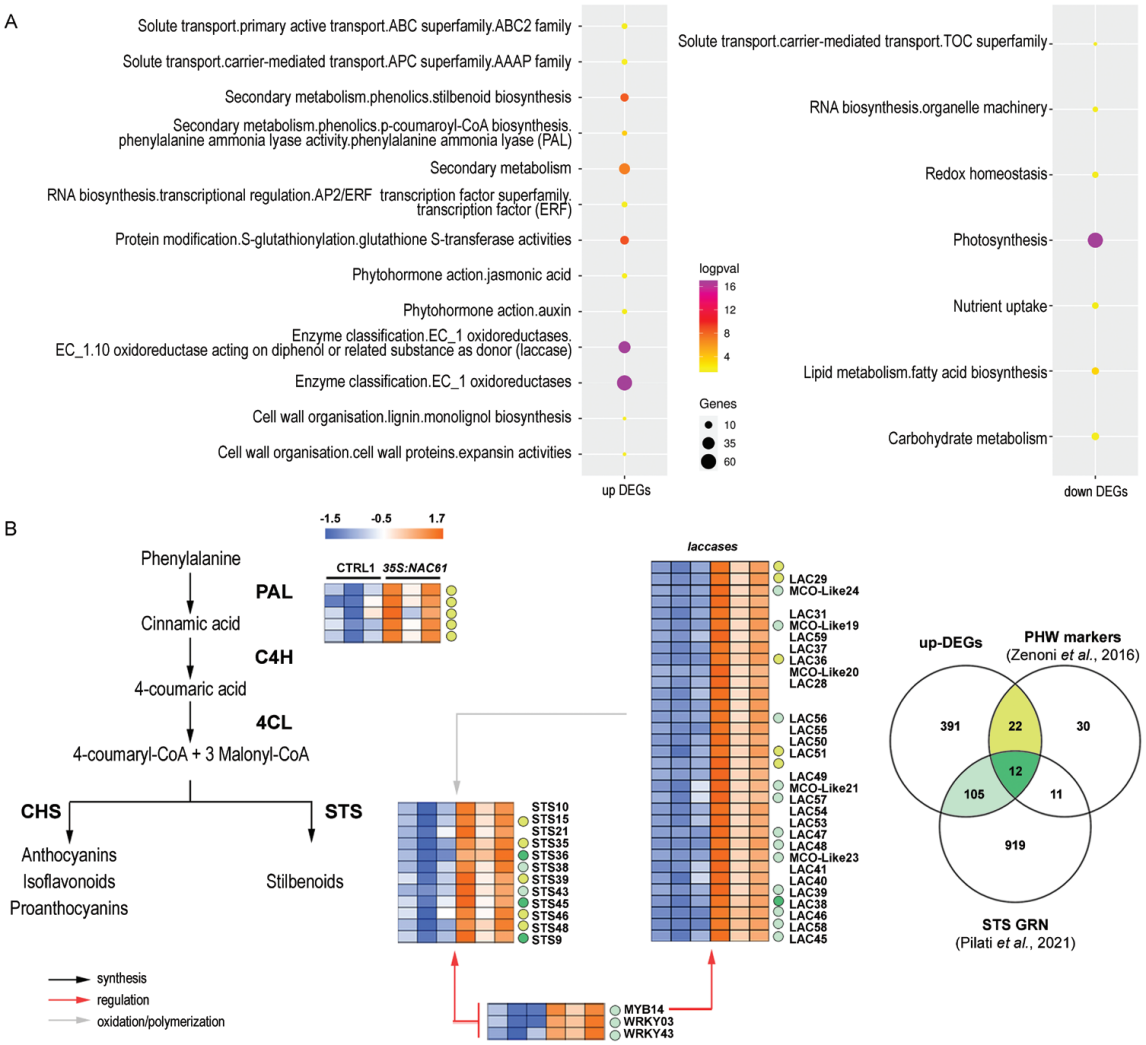
Transcriptomic responses to *NAC61* overexpression in leaves of grapevine cv. ‘Thompson seedless’. (A) Functional enrichment analysis of up-regulated and down-regulated DEGs. (B) Heatmap of up-regulated DEGs involved in phenylpropanoid synthesis, regulation, and modification (Supplementary Dataset S2). Markers of the PHW process (Zenoni *et al.*, 2016) and genes belonging to the *STS* GRN (Pilati *et al.*, 2021) are highlighted according to the color code in the Venn diagram.

Grapevine cv. ‘Thompson Seedless’ plants transiently overexpressing *NAC61* (Supplementary Fig. S4A, B) showed 1157 DEGs compared with control plants (Supplementary Dataset S2). Among the DEGs, 530 genes were up-regulated and 627 were down-regulated. As previously reported for *NAC33* and *NAC60* (D’Incà *et al.*, 2021, 2023), no clear phenotypic alterations were observed in *NAC61*-overexpressing ‘Thompson Seedless’ leaves. Gene category MapMan distribution and DEGs enrichment analysis reveal that up-regulated genes are mainly involved in ‘Secondary metabolism’, in particular ‘Stilbenoid biosynthesis’, and in ‘Oxidoreductases activity’, in particular ‘Laccases’, whereas down-regulated genes are mainly represented by ‘Photosynthesis-related mechanisms’ and other primary metabolism-related processes, such as ‘Lipid and carbohydrate metabolisms’ (Fig. 3A; Supplementary Dataset S3).

We highlight that NAC61 highly affects the expression of five *phenylalanine ammonia lyases* (*PALs*), corresponding to the first and committed step in the phenylpropanoid pathway, and 12 *STSs*, genes encoding the key enzymes leading to stilbenoid biosynthesis (Fig. 3B; Supplementary Dataset S2). The five *PALs* and eight of the 12 *STSs* were previously described as markers of post-harvest dehydration (Zenoni *et al.*, 2016). Up-regulated DEGs also included 33 *laccases* (*LACs*), which are proposed to be involved in the oxidative polymerization of phenolic compounds (Keylor *et al.*, 2015), six of which were also described as markers of post-harvest dehydration (Fig. 3B; Supplementary Dataset S2). Overall, 34 out of the 75 molecular markers of post-harvest berry dehydration were found to be up-regulated by NAC61 (Supplementary Dataset S2).

By inspecting the recently proposed *STS* gene regulatory network (GRN), built by the OneGenE tool (Pilati *et al.*, 2021), we found 117 of the 530 up-regulated genes (Fig. 3B; Supplementary Dataset S2), including the stilbenoid regulators *MYB14, WRKY03*, and *WRKY43*. Moreover, 13 out of all of the up-regulated *LACs* are also found in the *STS* GRN (Supplementary Dataset S2), indicating their potential role in stilbene polymerization (i.e. the production of viniferins), as previously suggested (Zenoni *et al.*, 2016; Pilati *et al.*, 2021; Orduña *et al.*, 2022). The up-regulation of *MYB14* and a *laccase* gene (*LAC25*; *VIT_18s0001g01280*) in the *NAC61-*overexpressing plants was validated by RT–qPCR (Supplementary Fig. S4C). Of note, the ectopic expression of *NAC61* led to the over-expression of *NAC61* itself.

### Examination of the NAC61 cistrome for identifying NAC61 high-confidence targets

**Fig. 4.**
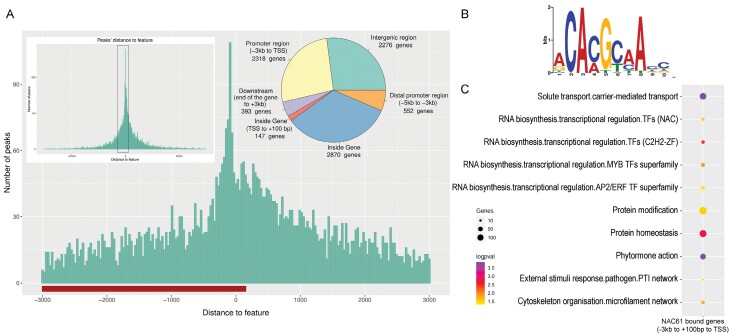
NAC61 DAP-seq analyses. (A) Distribution of NAC61 DNA-binding events with respect to their position from the TSS of their assigned genes. The distribution of peak positions is represented in the pie chart. (B) *De novo* forward binding motif obtained from the inspection of the top 600 scoring peaks of the NAC61 library using the RSAT tool. (C) Functional enrichment analysis of genes to which NAC61 bound (from –3 kb to +100 bp relative to the TSS).

To identify putative direct targets of NAC61, we inspected its genome-wide binding landscape (cistrome) by carrying out DAP-seq. We identified 8558 binding events assigned to 6734 genes (Fig. 4A). The distribution of NAC61 DNA-binding events, with respect to their position from the transcription start site (TSS) of the identified genes, showed a preferential localization in proximal upstream regions and inside genes (Fig. 4A; Supplementary Dataset S4). Within the ‘inside gene’ category, most binding events were found at the very start of the gene feature, that is, close to 100 bp. By inspecting the 600 top-scoring peaks, we identified the major binding motif [CA(C/A)G(C/T)(A/C)A] (Fig. 4B), correlated with *A. thaliana* ANAC46, which controls cell death during leaf senescence (Huysmans *et al.*, 2018), ANAC55, which is involved in ABA and jasmonic acid responses (Jiang *et al.*, 2009), and ANAC047, the closest homologue of NAC60 (D’Incà *et al.*, 2023) (Supplementary Fig. S5) according to RSAT Plants phylogenetic footprints. To identify the putative targets of NAC61, we focused on genes for which the TF bound to their promoter region (from –3 kb to +100 bp relative to the TSS), and identified 2471 peaks and 2263 unique genes (Supplementary Dataset S4). The use of MapMan ontology of these genes highlighted functional enrichment in the descriptors ‘Transcriptional regulation (*zinc fingers*, *NACs*, *MYBs*, *ERFs*)’, ‘Solute transport’, ‘Protein homeostasis’, and ‘External stimuli and pathogen response’ (Fig. 4C).

**Fig. 5.**
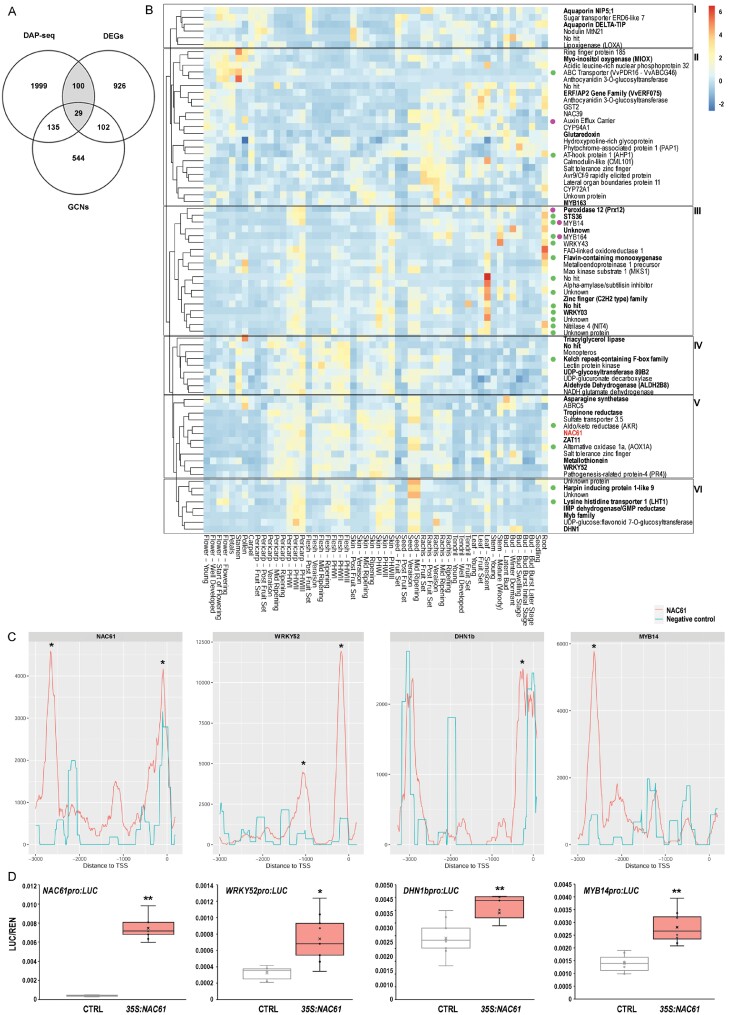
Identification and validation of HCTs. (A) Venn diagram showing the number of common genes between the DAP-seq bound genes (peaks in the region from –3 kb to +100 bp relative to the TSS), DEGs (FC ≥1.5 and adjusted *P*-value <0.1), and GCNs (berry, leaf, and tissue-independent datasets) (Supplementary Dataset S5). The NAC61 HCTs are in the grey-shaded sections. (B) Heatmap representing the atlas expression (Fasoli *et al.*, 2012) of the HCTs up-regulated by the overexpression of NAC61 in cv. ‘Thompson seedless’ leaves. The clusterization of HCTs was performed by using the Expression Atlases App (Corvina) within the VitViz platform (http://www.vitviz.tomsbiolab.com/), using the *z*-score data transformation and clustering by row. The 29 genes shared by the three datasets are highlighted in bold. Markers of the PHW process (Zenoni *et al.*, 2016) and genes belonging to the STS GRN (Pilati *et al.*, 2021) are indicated with violet and green circles, respectively. (C) NAC61 DNA-binding events shown as density plots and delimited between –3 kb and +100 bp from the TSS of *NAC61*, *WRKY52*, *DHN1b*, and *MYB14*. The peaks were identified by GEM and were pointed out with their corresponding signal score in the proximal promoter regions. Asterisks indicate the most significant peaks obtained by the DAP-seq analysis. The negative control corresponds to an input library generated with an empty GST-HALO vector. (D) *NAC61*, *WRKY52*, *DHN1b*, and *MYB14* promoter activation by NAC61 tested by DLRA in infiltrated *N. benthamiana* leaves. LUC values are reported relative to the REN value. Each value represents the mean ±SD of three biological replicates tested in technical replicate (*n*=3). Asterisks indicate statistically significant differences (*P<0.05, **P<0.01; *t*-test).

To define NAC61 high-confidence targets (HCTs), we then overlapped the 1157 cv. ‘Thompson Seedless’ DEGs and the 2263 NAC61-bound unique genes (Fig. 5A). A total of 129 HCTs were thus identified (29 of which were in common with at least one of the GCNs; Supplementary Dataset S5). Interestingly, *NAC61* itself and other 15 annotated TF genes were identified among the 78 HCTs, up-regulated in *NAC61*-overexpressing plants, and further assigned to six clusters according to their expression profile in the global gene expression atlas (Fasoli *et al.*, 2012; Fig. 5B). This allowed us to focus on the genes most closely correlated with *NAC61* throughout the development of different grapevine organs.

Among the HCTs belonging to the *NAC61* cluster (Cluster V), we found several candidate genes putatively involved in abiotic and biotic stress responses, such as two *zinc fingers* (*VIT_13s0019g00480*; *VIT_06s0004g04180*), an *aldo/keto reductase* (*AKR*; *VIT_05s0062g00980*), an *alternative oxidase* (*AOX1A*; *VIT_02s0033g01400*), *pathogenesis-related protein 4* (*PR4;VIT_14s0081g00030*), encoding a chitinase, and *WRKY52*, recently reported as a *Botrytis cinerea* susceptibility gene (Wang *et al.*, 2018). Albeit less closely correlated with NAC61 expression, other clusters included transcripts functionally associated with stress responses. In Cluster VI, we found a *harpin inducing protein 1-like 9* (*VIT_08s0007g02360*), a *lysine histidine transporter 1* (*LHT1*; *VIT_06s0061g01210*), and the previously described *DHN1b,* all highly expressed in berry during post-harvest dehydration and in seed after veraison. Moreover, the *aquaporins NIP5* (*VIT_02s0025g03260*) and *DELTA-TIP* (*VIT_09s0002g04020*), the *nodulin MtN21* (*VIT_01s0026g00520*), and a *lipoxygenase* (*LOXA; VIT_06s0004g01510*) were identified in Cluster I; a *myo-inositol oxygenase* (*MIOX*; *VIT_11s0016g02800*), a *glutathione S-transferase* (*GST2*; *VIT_07s0005g00030*), a *glutaredoxin* (*VIT_10s0003g00390*), a *calmodulin* (*CML101*; *VIT_01s0010g02930*), a *salt tolerance zinc finger* (*VIT_18s0001g09230*), and the *AVR9/*CF*-9 rapidly elicited protein* (*VIT_01s0011g06140*) were identified in Cluster II; and an *aldehyde dehydrogenase* (*ALDH2B8*; *VIT_01s0026g00220*), *NADH glutamate dehydrogenase* (*VIT_16s0039g02750*), *Monopteros* (*VIT_04s0043g00940*), a *triacylglycerol lipase* (*VIT_07s0005g01240*), and a *kelch repeat-containing F-box family protein* (*VIT_14s0068g02150*) were identified in Cluster IV. The above-described stilbenoid-related genes *STS36* (*VIT_16s0100g01100*), *MYB14*, *WRKY43*, and *WRKY03* are also found among the NAC61 HCTs. These genes, together with a *flavin-containing monooxygenase* (*VIT_07s0104g01260*), *MAP kinase substrate 1* (*MKS1*; *VIT_01s0011g03650*), *peroxidase 12* (*Prx12*; *VIT_18s0072g00160*), *nitrilase 4* (*NIT4; VIT_02s0109g00430*), and the *MYB164* (*VIT_17s0000g03560*), were characterized by an increase of expression in berry skin during post-harvest dehydration, in senescing leaf, and in root (Cluster III). The NAC61-binding signal found in the promoters of *WRKY52*, *DHN1b*, *MYB14*, and *NAC61* (Fig. 5C; Supplementary Fig. S6) was confirmed by DLRA, showing a significant activation by NAC61 (Fig. 5D).

### NAC61 self-activates and synergistically interacts with the grape berry ripening master regulator NAC60

**Fig. 6.**
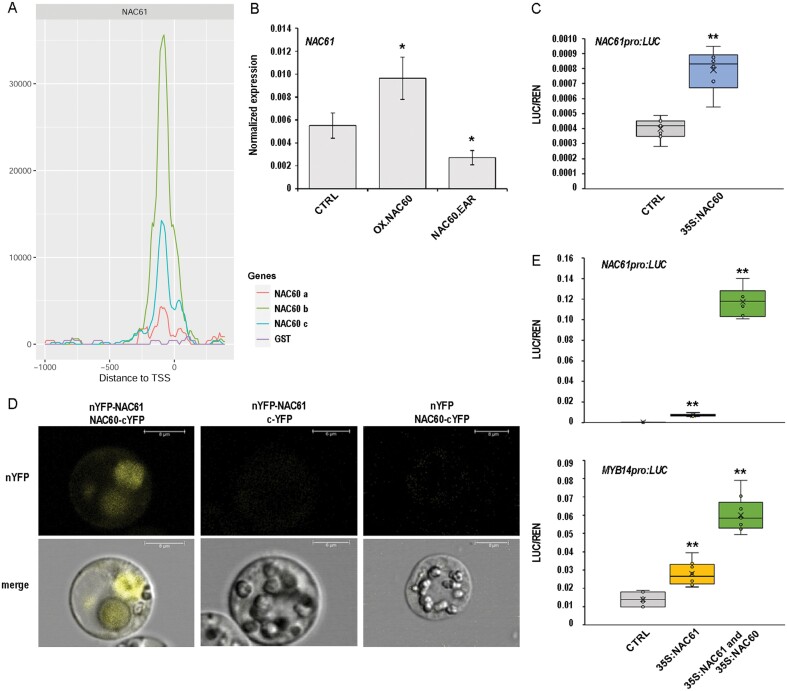
The NAC61–NAC60 regulatory complex regulates *NAC61* and *MYB14* activation. (A) NAC60 DNA-binding events shown as density plots and delimited between –3 kb and +100 bp from the TSS of *NAC61.* The NAC60 binding motifs were searched for in three different genomic libraries (a, berry gDNA; b and c are biological replicates of leaf gDNA; D’Incà *et al.*, 2023) (B) *NAC61* expression level in grapevine leaves stably overexpressing *NAC60* (OX:NAC60) and expressing the *NAC60* dominant repressor (NAC60.EAR), determined by RT–qPCR. Each value is relative to the expression of *UBIQUITIN1* (*VIT_16s0098g01190*) and represents the mean ±SD of three biological replicates (D’Incà *et al.*, 2023). Asterisks indicate statistically significant differences (*P<0.05; *t-*test) in comparison to the control. (C) *NAC61* promoter transactivation by NAC60 tested by DLRA in infiltrated *N. benthamiana* leaves. LUC values are reported relative to the REN value. Each value represents the mean ±SD of three biological replicates tested in technical replicate (*n=*3). Asterisks indicate statistically significant differences (**P<0.01; *t-*test). (D) BiFC analysis in grapevine protoplasts showing NAC61–NAC60 protein interaction. Corresponding controls are also shown. Images show a representative case of YFP signal being detected in the cell nucleus by confocal laser scanning. (E) *NAC61* and *MYB14* promoter activation tested by DLRA in infiltrated *N. benthamiana* leaves. The activity of NAC61 alone (also reported in Fig. 5D) and combined NAC61–NAC60 activity were tested. LUC values are reported relative to the REN value. Each value represents the mean ±SD of three biological replicates tested in technical replicate (*n=*3). Asterisks indicate statistically significant differences (**P<0.01; *t-*test). The data reported in C and E were derived from the same experiment and control values are therefore the same.

*NAC61* was recently identified as a putative target of NAC60 (Fig. 6A) (D’Incà *et al.*, 2023) and shows a delayed expression pattern in comparison to that of *NAC60* in developing berries (Fig. 1A). Here, we demonstrated that NAC60 directly controls the activation of NAC61 (i) by inspecting the expression of *NAC61* in previously produced transgenic grapevines with altered NAC60 activity and (ii) by DLRA. In comparison to the wild type, a significantly higher *NAC61* expression level was observed in leaves stably overexpressing *NAC60*, whereas a significantly lower *NAC61* expression level was observed in leaves expressing the *NAC60* dominant repressor to overcome endogenous NAC60 activity (Fig. 6B; Supplementary Fig. S7). A dominant repressor (also known as negative dominant) was created by fusing the NAC60 C-terminal to the plant-specific EAR repressor domain and placing this chimeric repressor under the control of the endogenous *NAC60* promoter (D’Incà *et al.*, 2023). Moreover, by performing DLRA, we demonstrated the ability of NAC60 to significantly transactivate (1.98-fold) the *NAC61* regulatory region (Fig. 6C).

Both *NAC61* and *NAC60* were found to be markers of the berry post-ripening phase (Zenoni *et al.*, 2016), and *NAC60* was also among the 92 common genes of the three *NAC61* GCN datasets. Using a BiFC assay, we demonstrated the physical interaction between the two NACs and found that a NAC60–NAC61 heterocomplex is localized into the nucleus (Fig. 6D). Accordingly, we also showed that NAC60 significantly increases the ability of NAC61 to activate itself (Figs 5C, 6E). Indeed, we found that *NAC61* is induced 18.57-fold by NAC61 alone, whereas the heterodimeric complex formed with NAC60 promotes a synergistic action which resulted in a 293.43-fold induction (Fig. 6E). Moreover, NAC60 was previously reported to activate *MYB14* alone (D’Incà *et al.*, 2023). Here, we found that NAC60 significantly increased the ability of NAC61 to activate the *MYB14* promoter (4.29-fold) in comparison to the action of NAC61 alone (2-fold) (Fig. 6E). By investigating the *NAC61* regulatory region (–3.0 kb to the TSS), we found two binding sites for *A. thaliana* ANAC047 (Supplementary Fig. S8), coinciding with the already identified NAC60-binding site and perfectly matching the NAC60-binding locations. We also found binding sites for RAP2.6, ABI3VP1/VRN1, and DEAR4, which are involved in ABA, osmotic, drought and salt stress responses (Q. Zhu *et al.*, 2010; J. Wang *et al.*, 2020; S. Wang *et al*., 2020; Zhang *et al.*, 2020), for RRTF1 and RAP2.3, which mediate plant defense responses against *B. cinerea* and other pathogens (León *et al.*, 2020; J. Wang *et al.*, 2020), for ORA47, a regulator of general stress responses induced by methyl jasmonate (Zeng *et al.*, 2022), and for DREB2C, AP2EREBP, and G2like_tnt.At3g13040, which are involved in response to drought and dehydration (Riechmann and Meyerowitz, 1998; Kizis *et al.*, 2001; Dietz *et al.*, 2010; Lee *et al.*, 2010; Wang *et al.*, 2022), corroborating the role of *NAC61* in abiotic and biotic stress responses (Supplementary Fig. S8).

### *NAC61* expression is enhanced by high temperature and *B. cinerea* infection during berry post-harvest dehydration

**Fig. 7.**
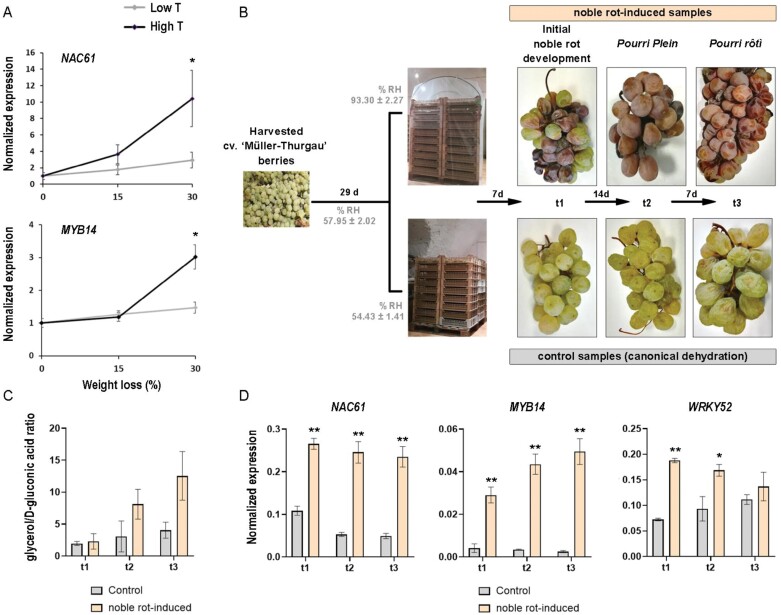
Trends in the expression of *NAC61* and target genes during post-harvest dehydration conducted in different conditions. (A) *NAC61* and *MYB14* expression levels during post-harvest dehydration performed under high- and low-temperature conditions (Shmuleviz *et al*., 2023). Each value is relative to the expression of *UBIQUITIN1* (*VIT_16s0098g01190*) and data presented are the mean ±SD of three biological replicates. Asterisks indicate significant differences (*P<0.05; *t-*test). (B) Experimental plan for noble rot induction. Berries of cv. ‘Müller Thurgau’ were collected at full maturity and put in a dehydrating room for 29 d to reach 30% weight loss. Then, half of the berries were covered to induce noble rot. The three stages (t0, t1, and t2) of infected and control berries collected for further analyses are shown with representative images. (C) Glycerol to d-gluconic acid ratio assessed as an indicator of noble rot development. Each value corresponds to the mean ±SD of three replicates. Asterisks indicate significant differences (*P<0.05; *t-*test). (D) *NAC61*, *MYB14*, and *WRKY52* expression levels in noble-rot-induced berries tested at different phases of *B. cinerea* infection in cv. ‘Müller-Thurgau’ berries (noble-rot-induced samples) compared with control berries. Each value is relative to the expression of *UBIQUITIN1* (*VIT_16s0098g01190*) and represents the mean ±SD of three biological replicates. Asterisks indicate statistically significant differences (*P<0.05, **P<0.01; *t*-test) of the noble-rot-induced samples compared with the controls.

Taking advantage of the experimental plan from a recent study (Shmuleviz *et al.*, 2023), we analyzed the expression of *NAC61* in grape berries subjected to post-harvest dehydration in two different temperature regimens. The results showed that *NAC61* expression is significantly induced by high temperature, similar to its HCT *MYB14* (Fig. 7A; Supplementary Fig. S9A).

Because previous reports suggested that *NAC61* is induced during grape infection by *B. cinerea*, in conditions of noble rot development but not of grey mold (Blanco-Ulate *et al.*, 2015; Kelloniemi *et al.*, 2015), we set up specific experimental conditions to induce noble rot in harvested berries (Negri *et al.*, 2017) and analyzed the expression of *NAC61* and some of its putative targets. Bunches of cv. ‘Müller-Thurgau’ were harvested at full maturity and placed in a ventilated dehydrating facility under controlled conditions (Fig. 7B). After 29 d of dehydration, when the soluble solid content reached ~24 °Brix, half of the bunches were covered with plastic film to naturally increase the RH, which is required for noble rot induction (Fig. 7B). In these conditions, dehydration and juice solute concentration were limited (Supplementary Fig. S7B, C), and the first signs of noble rot appeared on berries 7 d after coverage (t1). After a further 14 d, infected berries reached the characteristic *pourri plein* stage (t2), and 1 week later, the clearly shriveled berries reached the *pourri rôtì* stage (t3) (Fig. 7B) (Negri *et al.*, 2017). The glycerol to d-gluconic acid ratio was assessed as an indicator of noble rot development (Fig. 7C) (Ribéreau-Gayon *et al.*, 2006). The analysis of transcript levels by RT–qPCR in control and noble-rot-induced berries showed a strong up-regulation of *NAC61* and the NAC61 targets *MYB14* and *WRKY52* during noble rot development (Fig. 7D; Supplementary Fig. S9A).

## Discussion

### NAC61 controls the stilbenoid biosynthetic pathway as a conserved feature of late and post-ripening

Stilbenoids are a group of polyphenols synthesized by STSs in response to biotic, abiotic, and developmental cues. *STSs* are also expressed in the absence of external stimuli in a tissue- and cultivar-dependent manner (Versari *et al.*, 2001; Pezet *et al.*, 2003; Jean-Denis *et al.*, 2006; Gatto *et al.*, 2008; Eisenmann *et al.*, 2019). In grapevine, *STSs* represent a large gene family encompassing 41 isoforms (Vannozzi *et al.*, 2012), most of which are regulated by subgroup 2 R2R3-MYB TFs (i.e. MYB13/MYB14/MYB15; Orduña *et al*., 2022). Among the 12 transiently activated *STSs* in *NAC61*-overexpressing leaves (Fig. 3B), *STS36* (Huang *et al.*, 2018) has been identified as a candidate NAC61 HCT. Additionally, our results demonstrate that NAC61 binds to the regulatory regions of *MYB14* (which is also directly regulated; Fig. 5), *WRKY03* and *WRKY43*, all of which cooperate in enhancing *STSs* expression (Vannozzi *et al.*, 2018). These results allow us to place NAC61 in a high hierarchical position for the regulation of stilbene synthesis. Also corroborating this NAC61-dependent transcriptional cascade, 117 out of 530 genes up-regulated by *NAC61* overexpression belong to the recently described *STSs* GRN including more than 1000 structural and regulatory genes potentially involved in stilbenoid metabolism (Fig. 3B; Supplementary Dataset S2) (Pilati *et al.*, 2021). As well as the above-described HCTs (*STS36*, *MYB14*, *WRKY03*, and *WRKY43*), we identified two other R2R3-MYB members, *MYB163* and *MYB164*, previously associated with the phenylpropanoid pathway (Wong *et al.*, 2016).

In grapevine, LACs have been proposed to control the oxidative polymerization of both monomeric stilbenes and monolignols, producing viniferins and lignin, respectively (Keylor *et al.*, 2015). *Vitis vinifera* LAC family members were recently assigned to the stilbenoid- and lignin-related subgroups based on sequence similarity and co-expression (Pilati *et al.*, 2021). Interestingly, among the 33 *LACs* up-regulated in *NAC61*-overexpressing grapevine plants, 23 belong to the stilbenoid subgroup, whereas none of these belong to the lignin-related subgroup. Nevertheless, the absence of *LAC* genes among the HCTs indicates that NAC61-mediated *LAC* regulation may occur indirectly, likely through MYB14 as shown by Orduña *et al.* (2022).

Among the NAC61 HCTs, we also found *AKR*, *AOX1A*, *AT-hook protein 1* (*AHP1*), a *flavin-containing monooxygenase*, *harpin inducing protein 1-like 9*, *kelch repeat-containing f-box family protein*, and *LHT1* to be present in the *STSs* GRN (Supplementary Dataset S5). Although the role of these genes in stilbenoid metabolism, is not clear, and further investigations are needed, their belonging to the NAC61 HCTs strongly supports the master regulatory role of NAC61 in the synthesis and modification of stilbenes. The accumulation of stilbenoids is a hallmark of the late- and post-ripening stages; thus, the genes related to their synthesis can be considered true markers of these developmental transitions. Consistently, 34 out of the 75 PHW molecular markers defined by Zenoni *et al.* (2016) are up-regulated by *NAC61* overexpression, including *JAZ4*, eight STSs, six *LACs*, the *dirigent protein DIR16*, four *nitrilases*, an *osmotin*, *Prx12*, and a *pathogenesis-related protein*, in addition to the previously mentioned *MYB14*, *MYB164*, *WRKY03*, and *WRKY43* (Fig. 3B; Supplementary Dataset S2).

### NAC61 is responsive to osmotic stress

During the late- and post-ripening stages, grape berries are subjected to a progressive increase of solute concentration, resulting in a severe osmotic stress. Several pieces of evidence arising from our study strongly indicate the involvement of NAC61 in osmotic stress responses: (i) the close correlation between sugar concentration and *NAC61* expression; (ii) the earlier activation of *NAC61* in berry flesh, where sugars and other metabolites accumulate (in comparison to the skin); (iii) the higher RWC in *N. benthamiana NAC61*-expressing leaves in comparison to control leaves; and (iv) the identification of NAC61 HCTs potentially involved in the osmotic stress response through different strategies/mechanisms.

Four zinc finger protein-coding genes were found among the HCTs: two *salt tolerance zinc fingers*, a *zinc finger C2H2 type*, and the *C2H2-type zinc finger ZAT11*, belonging to the branch of the C2H2 family containing the ZAT domain, whose role in response to abiotic stresses in several plant species has been widely described (Liu *et al.*, 2022). Interestingly, *cis*-regulatory motifs of ZAT TFs were found in *dehydrin* (*DHN*) genes recently characterized in *Brachypodium* grasses (Decena *et al.*, 2021). *DHNs* are ubiquitously expressed in periods of low intracellular water content (Yang *et al.*, 2012; Liu *et al.*, 2017; Smith and Graether, 2022) and their role in coping with osmotic stress has been demonstrated in several species (Tiwari and Chakrabarty, 2021). In our study, we identified and validated *DHN1b* as a target of NAC61. *DHN1b* is up-regulated in withering grape berries (Zamboni *et al.*, 2008) and in leaves and berries subjected to water stress (Xiao and Nassuth, 2006; Savoi *et al.*, 2017). Further investigation to elucidate a putative cooperation of NAC61 and ZAT11 in regulating *DHN1b* expression in osmotic stress conditions would be worthwhile in future studies.

The NAC61 HCTs include also *LHT1,* a major candidate for root acquisition of aspartate and a transporter of aspartate, asparagine, and glutamate in rice (Guo *et al.*, 2020). In grapevine, the induction of a *lysine histidine transporter* and other amino acid transporters in salt-stressed plants has been observed (Aydemir *et al.*, 2020). This finding, together with the increased RWC in the *NAC61-*overexpressing *N. benthamiana* leaves, suggests that NAC61 could exert its role in the water/osmotic stress response by regulating amino acid accumulation. Accordingly, an *asparagine synthase* is the HCT most strongly up-regulated by *NAC61* overexpression. This enzyme catalyzes the synthesis of asparagine from aspartate, and asparagine is one of the most represented amino acids in grape berries (Bouloumpasi *et al.*, 2015). Together, these two amino acids are widely described as drought-responsive metabolites (Han *et al.*, 2021). In addition, a *glutamate dehydrogenase* (*GDH*), which catalyzes the oxidative deamination of glutamate to generate α-ketoglutarate (Sullivan *et al.*, 2015), thus providing the carbon for *de novo* synthesis of aspartate, was also identified among the NAC61 HCTs.

Osmotic stress responses could also involve auxin metabolism, which in turn may contribute to drought tolerance through regulation of stomatal closure. Interestingly, an *auxin efflux carrier* and the ARF TF *Monopteros*, which inhibits stomatal development (Zhang *et al.*, 2014), were found among the NAC61 HCTs. Similarly, the down-regulation of the HCTs *histidine phosphotransfer AHP4*, whose knockout narrows stomatal apertures, heightens leaf temperatures during water stress, and increases leaf RWC (Ha *et al.*, 2022), and the *phosphatase PP2CA/AHG3*, whose repression activates ABA-mediated signaling pathway leading to stomatal closure and water retention (Jung *et al.*, 2020), may contribute to NAC61 function in osmotic stress responses. Interestingly, hypoxia-related genes are found among *NAC61-*induced DEGs, such as a *Hypoxia-responsive gene* and three dehydration-responsive proteins (*RD22*), indicating a decrease in oxygen supply, likely due to water saturation of the apoplast (van den Dries *et al.*, 2013).

The remodeling of lipid composition, to maintain the fluidity and stability of cell membranes, is another change adopted by plants to respond to osmotic stress. In this regard, among the HCTs, we found a *triacylglycerol lipase,* whose induction in grape berry under water deficit was previously reported (Savoi *et al.*, 2017). During prolonged drought, the membranes could be subjected to degradative processes due not only to lipolytic activity but also peroxidative activity. In this context, we could hypothesize an involvement of two NAC61 HCTs, *Prx12* and *LOXA*, both of which are also known to be involved in pathogen responses. The activity of these two enzymes could be related to the cell death observed in *NAC61*-overexpressing leaves 3 d post-agroinfiltration.

### NAC61 regulates redox state and defense genes during noble rot development

The significant increase of H_2_O_2_ (indicated by DAB staining) observed in *NAC61*-overexpressing *N. benthamiana* leaves and the NAC61-mediated up-regulation of five *Peroxidases* (*Prxs*), including the HCT *Prx12*, may account for apoplastic ROS production (Survila *et al.*, 2016). This strongly suggests a direct involvement of NAC61 in ROS accumulation. Interestingly, *Prxs* are a well-known class of pathogen-related (PR) proteins induced in host plant tissues by pathogen infection (Lüthje and Martinez-Cortes, 2018), suggesting a direct involvement of NAC61 in biotic stress responses as well. Accordingly, besides genes related to stilbenoid synthesis and osmotic stress that may account for grapevine defense against pathogens (Yang *et al.*, 2012), we also found several other HCTs related to biotic stress responses, such as *PR4*, the biotic-stress-responsive calmodulin-like *CML101* (Vandelle *et al.*, 2018), the *harpin inducing protein 1-like 9* and *AKR*, also identified as NAC60 targets (D’Incà *et al.*, 2023), an *Avr9/*Cf*-9 rapidly elicited protein*, *LOXA*, and *MKS1*.

The up-regulation of genes encoding PR proteins has been previously evidenced in healthy berries during PHW as part of a general response to biotic stresses (Zenoni *et al.*, 2016). We could then hypothesize that NAC61 is involved in ROS metabolism/homeostasis, on the one hand, by regulating the expression of genes involved in defense, while also activating mechanisms for ROS detoxification. Indeed, several genes involved in ROS scavenging/detoxification were found to be HCTs, such *MIOX*, a *flavin-containing monooxygenase*, *AOX1A*, an *aldehyde dehydrogenase (ALDH288*), a *glutaredoxin*, a *GDH*, and *GST2* (Zhang *et al.*, 2012; Munir *et al.*, 2020; Vanlerberghe *et al.*, 2020; Wang *et al.*, 2023).

ROS are also crucial signals for the induction of the hypersensitive response, a programmed cell death process that facilitates plant infection by necrotrophic pathogens (Soosaar *et al.*, 2005), including *B. cinerea* (Govrin and Levine, 2000), which is responsible for grey mold in grapes. However, in particular conditions, infection with the fungus leads to the development of noble rot, which promotes biochemical and metabolic changes in grape berries associated with interesting organoleptic features conferred upon sweet white wines (e.g. Amarone and Sauternes wines) thanks to a weaker (or even controlled) infection. The strong induction of *NAC61* in botrytized berries, together with the HCT *WRKY52*, which has been characterized as a grapevine susceptibility gene of *B. cinerea* (Wang *et al.*, 2018), indicates a possible direct involvement of *NAC61* in providing favorable conditions for noble rot development. This hypothesis is further supported by the identification among the HCTs of *MKS1,* whose overexpression in *A. thaliana* was shown to increase susceptibility to *B. cinerea* (Petersen *et al.*, 2010) and to promote the up-regulation of *Prx12* specifically during noble rot (Blanco-Ulate *et al.*, 2015; Lovato *et al.*, 2019). Moreover, *VqSTS36* was shown to enhance susceptibility to *B. cinerea* in *A. thaliana* and tomato (Huang *et al.*, 2018). On the other hand, NAC61 could also contribute to a weaker infection by simultaneously mitigating the favorable conditions for *B. cinerea* growth through the regulation of *PR4*, which encodes a chitinase, which could inhibit the growth of fungal hyphae (Grover, 2012), and *LOXA*, which is involved in the biosynthesis of jasmonate, known to mediate defense against necrotrophic pathogens (Antico *et al.*, 2012). Furthermore, in *A. thaliana* the overexpression of *JAZ8* represses defense responses against *B. cinerea* through its interaction with AtWRKY75 (Chen *et al.*, 2020). Considering that *WRKY52* is one of the closest homologues of *AtWRKY75* (Vannozzi *et al.*, 2018), and that two *JAZs*, namely *JAZ2* and *JAZ4*, are up-regulated by NAC61, a similar mechanism that allows noble rot development could be suggested in grape berries.

Interestingly, *Botrytis elliptica* infection of *Lilium regale* down-regulates *miR164* (Gao *et al.*, 2017), and the transient overexpression of *miR164f* in apple leaves enhances their susceptibility to *Alternaria alternata* AP, possibly due to the down-regulation of a *NAC* TF (Zhou *et al.*, 2023). Moreover, several NAC members belonging to the same clade as NAC61 are post-transcriptionally regulated by the *miR164* family (Kim *et al.*, 2009; Sun *et al.*, 2012; Lira *et al.*, 2017). We could therefore assume a scenario in which *B. cinerea* may affect *miR164* expression in the late berry ripening stage to allow an increase in the *NAC61* transcript level. Similarly, low-temperature storage conditions, which ultimately lead to a delay in fruit senescence, repress the strawberry genes *FaNAC087* and *FaNAC038* due to an increase of their negative regulator *miR164* (Xu *et al.*, 2013; Li *et al.*, 2017). Since *NAC61* also shows a lower expression in berries experiencing post-harvest dehydration under low-temperature conditions, control by *miR164* might be conserved in grape.

### NAC-dependent transcriptional network behind berry aging and stress responses

**Fig. 8.**
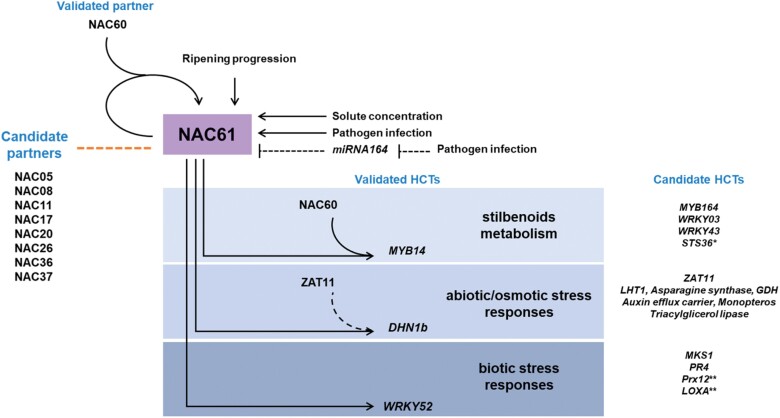
Proposed model of NAC61 mechanism of action. NAC61 high-confidence targets (HCTs) related to stilbenoid metabolism and stress responses that are inherent in late- and post-ripening phases are highlighted. The regulatory mechanisms controlling *NAC61* expression are also depicted. Validated mechanisms of transcriptional regulation are shown with black solid lines and hypothetical mechanisms are shown with dotted lines. Asterisks indicate genes putatively also involved in biotic stress responses (*) and abiotic/osmotic stress responses (**). The orange dotted line represents hypothetical physical interactions.

The GCNs obtained from different grapevine organs revealed a strong correlation between *NAC61* and many genes previously associated with the late- and post-ripening developmental stages (Supplementary Dataset S1). Consistently, NAC61 HCTs include several genes involved in stilbenoid metabolism as well as osmotic and biotic stress responses, which characterize these late processes (Fig. 8), thus suggesting NAC61 as a key regulator triggering the molecular mechanisms controlling ripening progression.

Interestingly, NAC60, which belongs to the *NAC61* GCN, not only activates the expression of NAC61 but also forms heterodimers with it, providing a mechanism for the transactivation of their common target *MYB14*. Moreover, the NAC61 self-activation is greatly increased by a proposed NAC60–NAC61 heterodimer (Fig. 8). These pieces of evidence, together with previously described NAC60–NAC03, NAC60–NAC33, and NAC33–NAC03 interactions, suggest that NAC61 participates in a NAC–NAC regulatory network, whose mechanism of action and additional players have just begun to be elucidated.

In addition, other *NACs* are co-expressed with *NAC61*, and thus represent putative partners. These include *NAC11*, previously described as a berry ‘switch gene’ by Massonnet *et al.* (2017), *NAC17*, which is involved in salinity and drought stress responses (Ju *et al.*, 2020), and *NAC26*, which has been proposed as a determinant of berry size variation (Tello *et al.*, 2015; Muñoz-Espinoza *et al.*, 2020) and a regulator of seed and fruit development through the interaction with MADS9 (Zhang *et al.*, 2021). Therefore, further lines of research should focus on the characterization of NAC61 downstream target genes and interacting proteins to elucidate the molecular mechanisms underlying berry aging and stress responses.

## Supplementary data

The following supplementary data are available at *JXB* online.

Fig. S1. *NAC61* expression pattern during berry development.

Fig. S2. Gene Ontology (GO) enrichment of the *NAC61* co-expressed genes.

Fig. S3. NAC61-containing cluster in the NACs phylogenetic tree.

Fig. S4. *NAC61* overexpression in cv. ‘Thompson Seedless’ grapevine leaves.

Fig. S5. NAC61 binding motif discovery analysis and motif comparison with published *A. thaliana* datasets.

Fig. S6. Scheme of the promoter regions amplified for the *NAC61*, *DHN1b*, *MYB14* and *WRKY52* transient activation experiment (Fig. 5D).

Fig. S7. Linear regression between the *UBIQUITIN1* and *EF1* expression level in the cv. ‘Syrah’ grape samples.

Fig. S8. *NAC61* regulative region analysis for *A. thaliana* ANAC047 and stress-related proteins *cis*-elements performed with the RSAT software.

Fig. S9. Transcriptomic and technological details on post-harvest withering grape sample.

Table S1. List of used primers.

Dataset S1. *NAC61* co-expressed genes. The GCNs were obtained separately by

Dataset S2. Transcriptomic analysis of *NAC61-*overexpressing and control cv. ‘Thompson Seedless’ leaves.

Dataset S3. Gene category MapMan distribution and enrichment analysis of DEGs.

Dataset S4. NAC61 DAP-seq bound genes.

Dataset S5. List of defined HCTs and detail of genes grouped in Fig. 5A.

erad507_suppl_Supplementary_Tables_S1_Figures_S1-S9

erad507_suppl_Supplementary_Datasets_S1

erad507_suppl_Supplementary_Datasets_S2

erad507_suppl_Supplementary_Datasets_S3

erad507_suppl_Supplementary_Datasets_S4

erad507_suppl_Supplementary_Datasets_S5

## Data Availability

Microarray data for the transient expression experiments on *V. vinifera* cv. ‘Thompson Seedless’ are available at GEO under accession no. GSE232165. DAP-seq raw data have been submitted to GEO, including metadata of samples and conducted analysis (bioinformatic parameters) according to the FAIR principles, under accession no. GSE230185. DAP-seq results on NAC61 can be visualized in the DAPBrowse tool available at the Vitis Visualization Platform (http://www.vitviz.tomsbiolab.com/). The role for NAC61 has been deposited in the Gene Reference Catalogue found at the Grape Genomics Encyclopedia portal (http://grapedia.org/). All other data supporting the findings of this study are available within the paper and within its supplementary data published online.
