# Supplementary material for: NAC61 regulates late- and post-ripening osmotic, oxidative, and biotic stress responses in grapevine
Source: J Exp Bot. 2023 Dec 30;75(8):2330–50. doi: 10.1093/jxb/erad507 (PMC11016852; doi:10.1093/jxb/erad507)
Supplement: erad507_suppl_Supplementary_Tables_S1_Figures_S1-S9 [file erad507_suppl_supplementary_tables_s1_figures_s1-s9.pdf]

Supplementary Figure S1. *NAC61* expression pattern during berry development. (A) *NAC61* expression profile in 10 different grapevine varieties at two pre- (pea and touch) and two post- (soft and harvest) veraison developmental stages (Massonnet *et al.*, 2017). Sampling time points are represented according to the BBCH scale for grapevine phenological growth stages as defined by Lorenz *et al*. (1995). (B) *NAC61* expression profile in cv. 'Cabernet Sauvignon' and 'Pinot noir' berries sampled every 10 days from fruit set to ripening (Fasoli *et al.*, 2018). (C) *NAC61* cluster of gene expression in the Genotype x Environment (GxE) dataset and box plot of Variable Importance Measure (VIM) used to characterize the relationship between the cluster and the experimental conditions (Dal Santo *et al.*, 2018). Sampling time points are represented according to the BBCH scale for grapevine phenological growth stages as defined by Lorenz *et al*. (1995). (D) *NAC61* expression trend in cv. 'Corvina' berries during traditional long and forced short post-harvest dehydration processes (Zenoni *et al.*, 2020). Weight loss percentage is specified for each point.

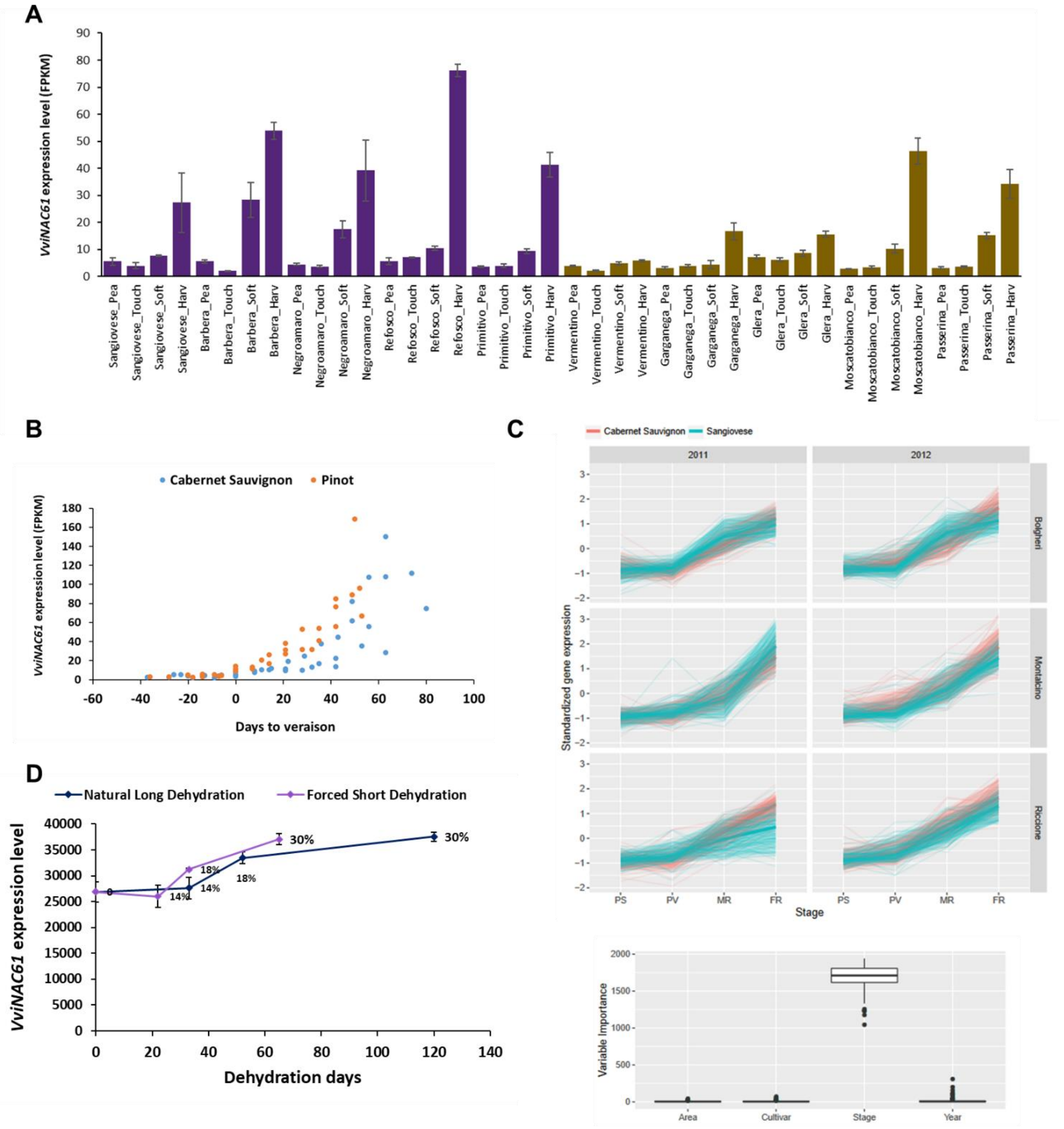
A
VviNAC61 expression level (FPKM)
Sangiovese_Pea
Sangiovese_Touch
Sangiovese_Soft
Sangiovese_Harv
Barbera_Pea
Barbera_Touch
Barbera_Soft
Barbera_Harv
Negroamaro_Pea
Negroamaro_Touch
Negroamaro_Soft
Negroamaro_Harv
Refosco_Pea
Refosco_Touch
Refosco_Soft
Refosco_Harv
Primitivo_Pea
Primitivo_Touch
Primitivo_Soft
Primitivo_Harv
Vermentino_Pea
Vermentino_Touch
Vermentino_Soft
Vermentino_Harv
Garganega_Pea
Garganega_Touch
Garganega_Soft
Garganega_Harv
Glera_Pea
Glera_Touch
Glera_Soft
Glera_Harv
Moscatobianco_Pea
Moscatobianco_Touch
Moscatobianco_Soft
Moscatobianco_Harv
Passerina_Pea
Passerina_Touch
Passerina_Soft
Passerina_Harv
B
Cabernet Sauvignon
Pinot
VviNAC61 expression level (FPKM)
Days to veraison
C
Cabernet Sauvignon
Sangiovese
2011
2012
Bolgheri
Montalcino
Riccione
Standardized gene expression
PS
PV
MR
FR
Stage
Variable Importance
Area
Cultivar
Stage
Year
D
Natural Long Dehydration
Forced Short Dehydration
VviNAC61 expression level
Dehydration days
14%
18%
30%

Supplementary Figure S2. Gene Ontology (GO) enrichment of the *NAC61* co-expressed genes. The GO enrichment analysis of the 810 genes co-expressed with *NAC61* (**Supplementary Dataset S1**) was performed by using the ShinyGO v.0.741 software (Ge *et al.*, 2020) with a False Discovery Rate (FDR) cutoff of 0.001.

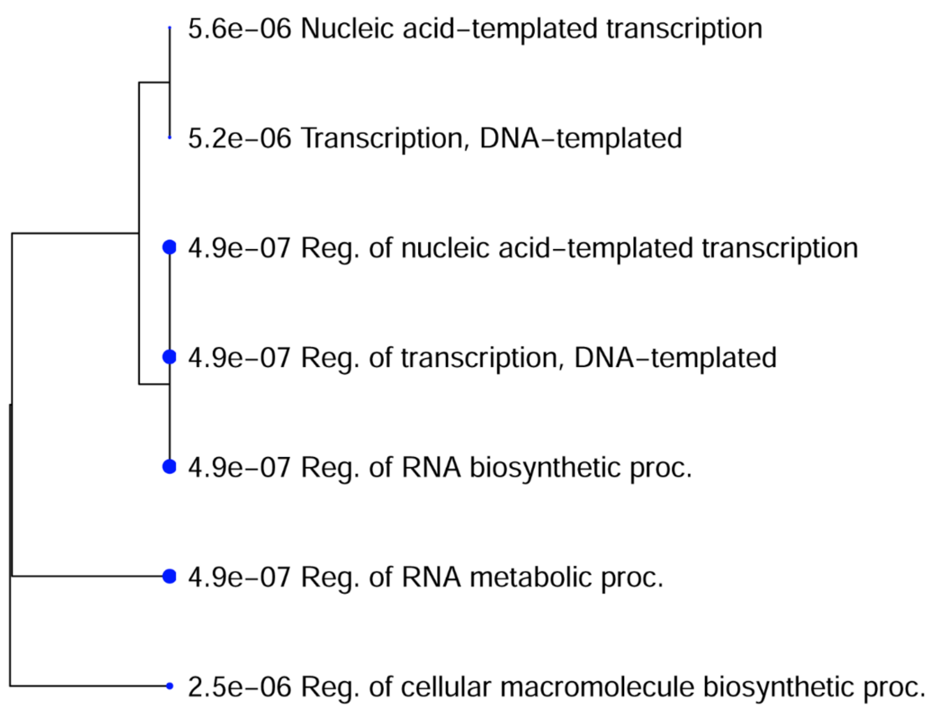

Supplementary Figure S3. NAC61-containing cluster in the NACs phylogenetic tree. Phylogenetic relationships of different plant species NAC genes were previously investigated (https://tomsbiolab.com/wp-content/uploads/2021/10/Fig.-S4.png; D’Incà *et al.*, 2023) and the NAC61-containing cluster is here reported. Red rows indicate ANAC046, the VvNAC61 closest homolog, VviNAC61 and VviNAC33.

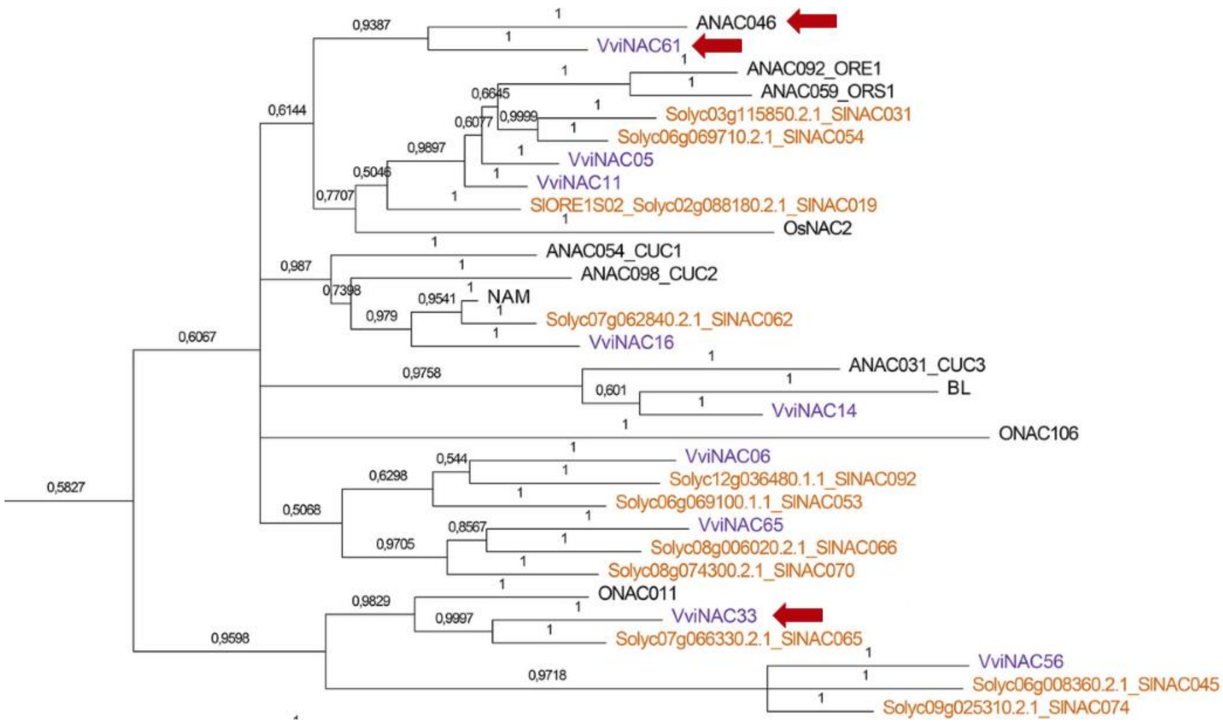

Supplementary Figure S4. *NAC61* overexpression in cv. 'Thompson Seedless' grapevine leaves. (A) The significant *NAC61* transient overexpression (*, p< 0.01; t-test) in comparison to the control was validated by RT-qPCR. Each value corresponds to the mean ± SD of three technical replicates relative to the *UBIQUITIN1*. (B) Linear regression between the normalized *NAC61* expression level using the *UBI1* (x axis) and the *EF1* (y axis) internal control, in the cv. 'Thompson seedless' leaf samples. (C) Expression level of *MYB14* and of a *laccase* gene (*VIT_18s0001g01280*) in *NAC61* overexpressing and in control lines determined by RT-qPCR. Each value corresponds to the mean ± SD of three biological and three technical replicates relative to the *UBIQUITIN1*. Asterisks indicate statistically significant differences (*, p< 0.01; t-test).

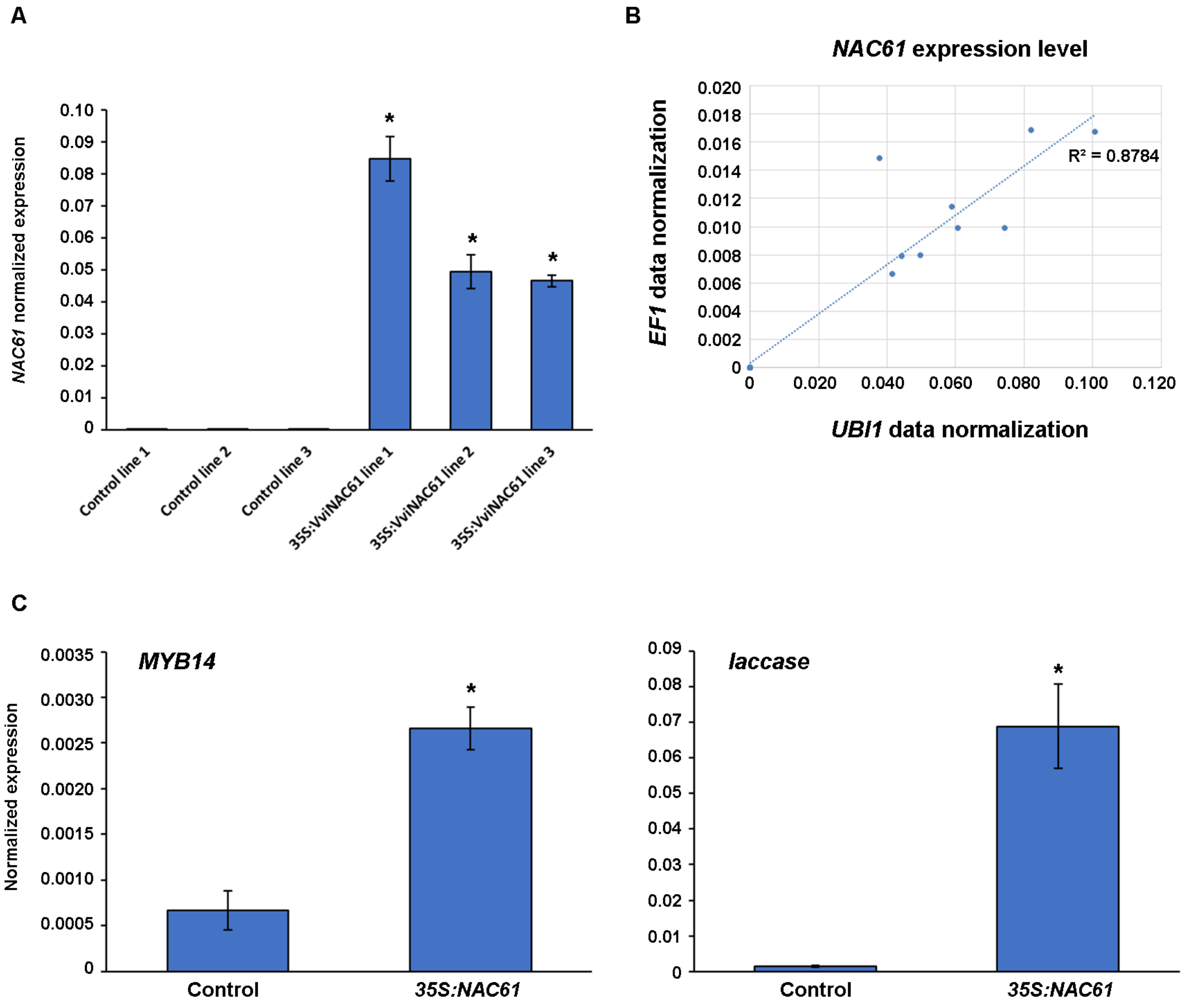

Supplementary Figure S5. NAC61 binding motif discovery analysis and motif comparison with published *A. thaliana* datasets.

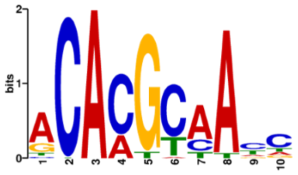

k-mer sig = **55.08**; evalue = **8.3e-56**

ArabidopsisPBM

Name	ID	Strand	Nb overlap columns	% aligned	Pearson correlation	Normalized cor
7195_ANAC58_ArabidopsisPBM_20140210_	7195_ANAC58_ArabidopsisPBM_20140210_	D	10	0.6250	0.943	0.590
7192_ANAC46_ArabidopsisPBM_20140210_	7192_ANAC46_ArabidopsisPBM_20140210_	D	10	0.6250	0.901	0.563
7193_ANAC55_ArabidopsisPBM_20140210_	7193_ANAC55_ArabidopsisPBM_20140210_	D	10	0.6250	0.860	0.537

Total matches = 3

Athamap

Name	ID	Strand	Nb overlap columns	% aligned	Pearson correlation	Normalized cor
No matches	No matches	No matches	No matches	No matches	No matches	No matches

Total matches = 0

Cistrome

Name	ID	Strand	Nb overlap columns	% aligned	Pearson correlation	Normalized cor
NAC_tnt.ANAC047_col_m1	NAC_tnt.ANAC047_col_m1	R	14	0.8235	0.809	0.666
NAC_tnt.NAM_colamp_a_m1	NAC_tnt.NAM_colamp_a_m1	D	11	0.6875	0.878	0.604
NAC_tnt.NAP_col_v3a_m1	NAC_tnt.NAP_col_v3a_m1	D	11	0.6875	0.874	0.601

Total matches = 41 (38 more)

Supplementary Figure S6. Scheme of the promoter regions amplified for the *NAC61*, *DHN1b*, *MYB14* and *WRKY52* transient activation experiment (**Fig. 5D**).

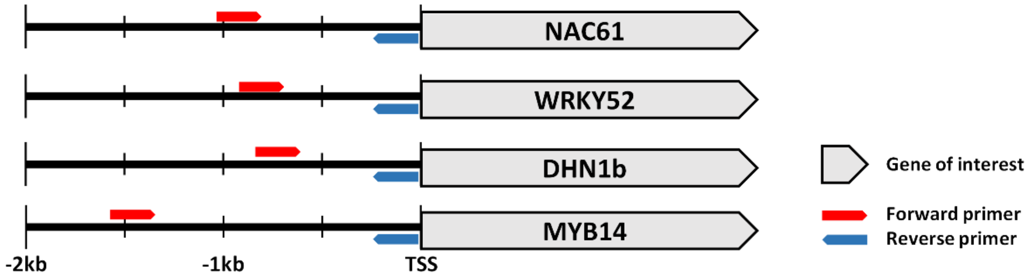

Supplementary Figure S7. Linear regression between the normalized *NAC61* expression level using the *UBI1* (x axis) and the *EF1* (y axis) internal control, in the cv. 'Syrah' leaf samples.

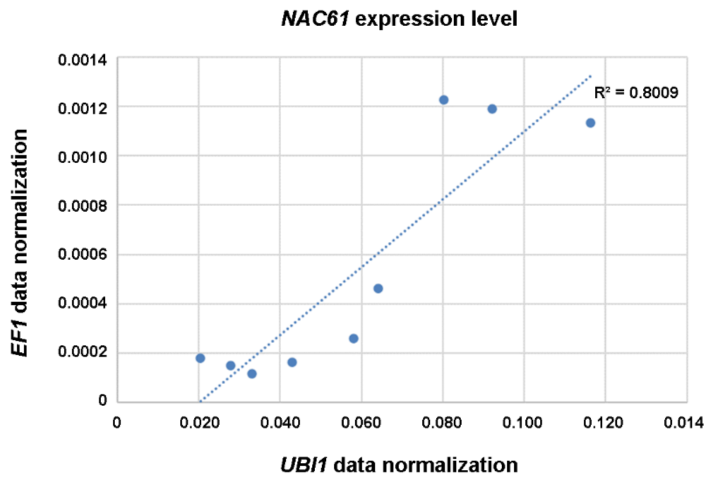

Supplementary Figure S8. *NAC61* regulative region analysis for *A. thaliana* ANAC047 and stress-related proteins *cis*-elements performed with the RSAT software. ANAC047, the NAC60 closest homolog, ORA47, RAP2.6 and RAP2.3, RRTF1, DEAR4, DREB2C, AP2EREBP, ABI3VP1 and G2like binding locations are reported.

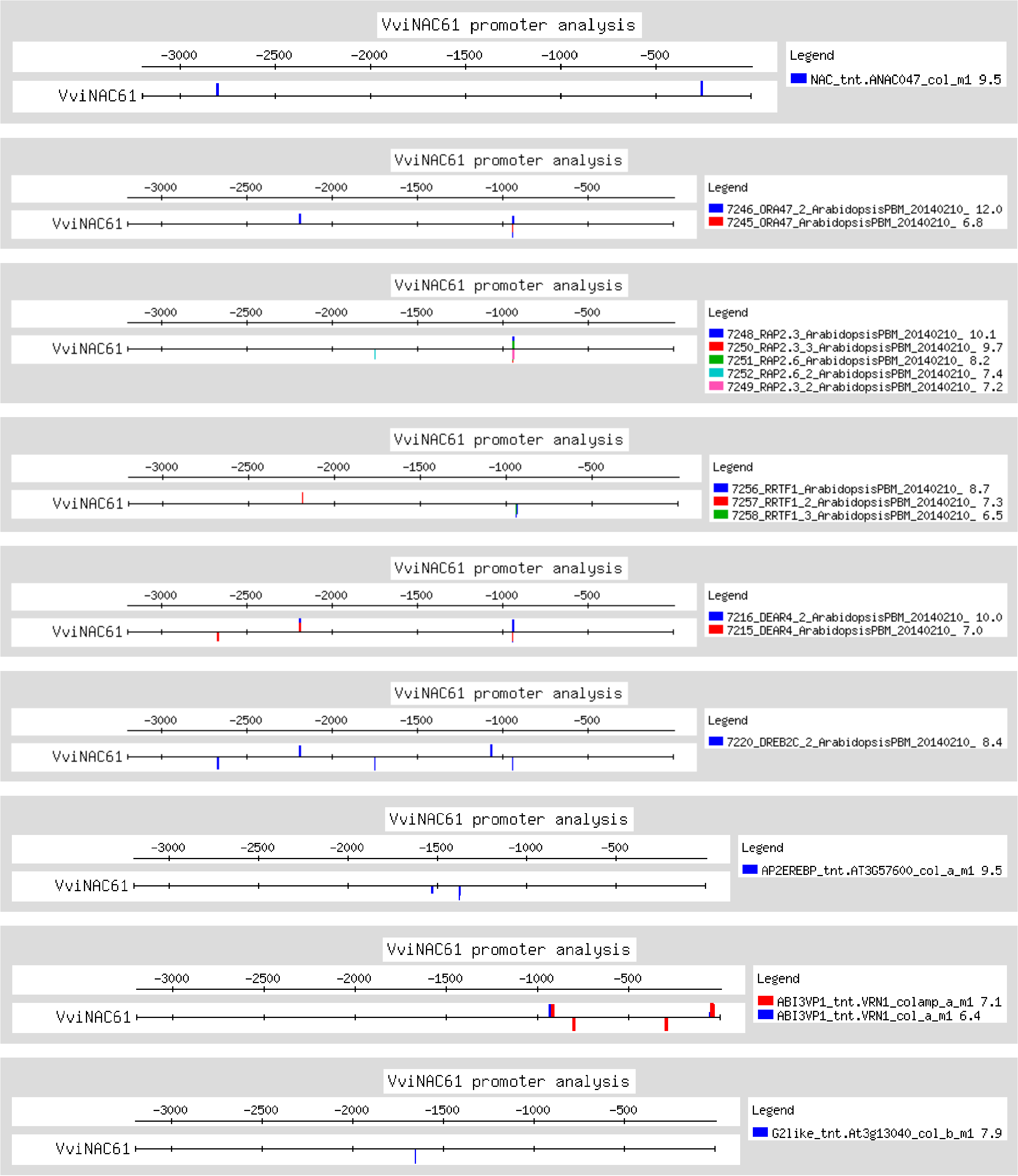

Supplementary Figure S9. Transcriptomic and technological details on post-harvest withering grape sample. Linear regression between the normalized *NAC61* expression level using the *UBI1* (x axis) and the *EF1* (y axis) internal control, in the cv. 'Corvina' (left) and cv. 'Müller Thurgau' (right) grape samples. (B) Sugar content (Brix°) and (C) Titratable Acidity (TA) trend in noble rot-induced and control cv. 'Müller Thurgau' grape berries throughout the post-harvest withering process; t1, t2 and t3 correspond to the three time points of grapes collection (**Fig. 7B**). Each value corresponds to the mean ± SD of three biological replicates.

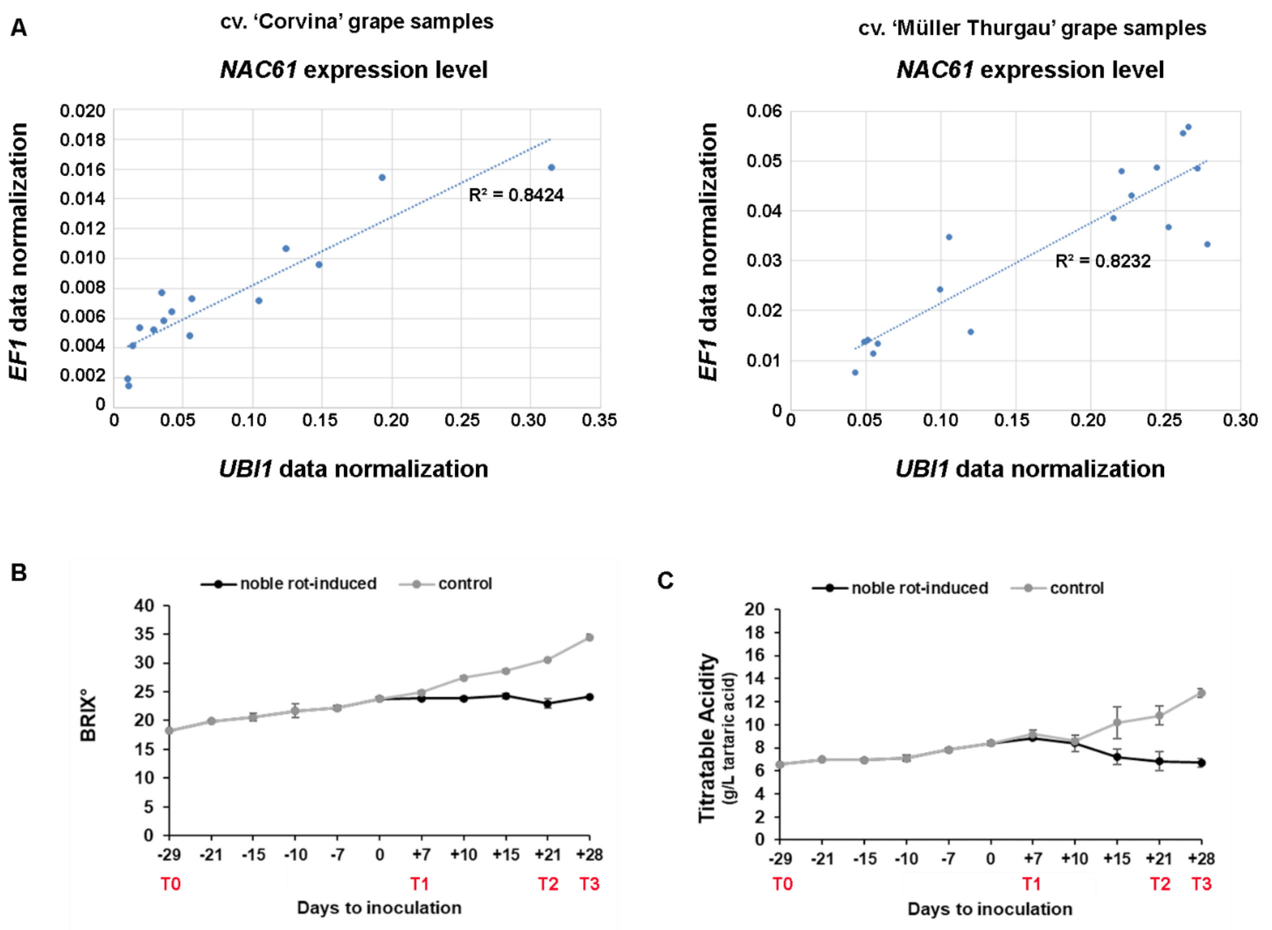

Supplementary Table S1. List of used primers.

Gene ID/Accession number	Functional annotation	Type of amplification	Primers (5'-3')		Ta (°C)	Amplicon (bp)
VIT_08s0007g07640	***NAC61***	CDS isolation	For	CACCATGGAAGAGGCTTCACTTG	55	1212
			Rev	TCAGTAGTCGAGTAAATAATCCA		
		Promoter isolation	For	CACCAATCTCTCTGAAAGCGGGGC	53	1012
			Rev	GGTTACACCACTACTGTATATCA		
		RT-qPCR	For	CGACCAGTACCGTAAGAGCC	55	93
			Rev	GCGAGAGGCTGACCATAGAC		
VIT_07s0005g03340	***MYB14***	Promoter isolation	For	CACCGGCTTCACCAATCATAGAGCTTA	55	1569
			Rev	TTTTTCTTTTCTACGTAAGGA		
		RT-qPCR	For	GATGTGGGAAATGGGAAATG	54	150
			Rev	CATTTGGCTGAGTCTGTTCG		
VIT_04s0023g02480	***DHN1b***	Promoter isolation	For	CACCCCCAACCACTCCACTACCAG	54	814
			Rev	TGTGTTGAAACGATCGATGAAATTT		
VIT_17s0000g01280	***WRKY52***	Promoter isolation	For	CACCTTGGTACACCACAAACGCAC	54	938
			Rev	TAGAGAGACTGAGAGAGATTGAGATTA		
		RT-qPCR	For	GAGTGGTGGACACCCATATCA	56	102
			Rev	AGTGATCATATCACAAGATCCTCCA		
VIT_18s0001g01280	***LACCASE***	RT-qPCR	For	TCACAGTGATTGGACCCGAA	57	188
			Rev	AATCAGAGGCATTGGGGTCA		
VIT_16s0098g01190	***UBIQUITIN1***	RT-qPCR	For	TCTGAGGCTTCGTGGTGGTA	55	99
			Rev	AGGCGTGCATAACATTTGCG		
VIT_12s0035g01130	***ELONGATION FACTOR1***	RT-qPCR	For	CAAGAGAAACCATCCCTAGCTG	55	91
			Rev	TCAATCTGTCTAGGAAAGGAAG		
AAN73054.1	***N.Benthamiana ACTIN gene***	RT-PCR	For	CTTGAAACAGCAAAGACCAGCTC	55	150
			Rev	TGCCAGCAGCTTCCATTCC		

Supplementary Dataset S1. *NAC61* co-expressed genes. The GCNs were obtained separately by looking at a berry-specific, a leaf-specific and a tissue-independent (TI) dataset. Key berry ripening regulators and DEGs during post-harvest dehydration are indicated.

Supplementary Dataset S2. Transcriptomic analysis of *NAC61*-overexpressing and control cv. 'Thompson Seedless' leaves. In sheet 2 and sheet 3 the upregulated and downregulated DEGs are reported, respectively. In sheet 2 berry post-harvest dehydration markers (Zenoni *et al*., 2016) and genes belonging to the STS GRN (Pilati *et al*., 2021) are reported.

Supplementary Dataset S3. Gene category MapMan distribution and enrichment analysis of DEGs.

Supplementary Dataset S4. NAC61 DAP-seq bound genes. In sheet 2 the promoter peaks selection (-3000≥bp ≥+100) is reported.

Supplementary Dataset S5. List of defined HCTs and detail of genes grouped in Fig. 5A. Genes belonging to NAC61 co-expressed genes (**Supplementary Dataset S1**), STS GRN (Pilati *et al.*, 2021), NAC60 VHCT (D'Incà *et al.*, 2023), and PHW markers (Zenoni *et al.*, 2016) are indicated. In sheets from 2 to 4 the DAP-seq, DEGs and GCNs exclusive genes are respectively reported; the common genes between 'GCNs and DAP-seq' and 'GCNs and DEGs' are reported in sheet 5 and 6, respectively; the DAP-seq, DEGs and GCNs common genes are listed in sheet 7.

Supplementary references

Lorenz DH, Eichhorn KW, Bleiholder H, Klose R, Meier U, Weber E. 1995. Growth Stages of the Grapevine: Phenological growth stages of the grapevine (Vitis vinifera L. ssp. vinifera)—Codes and descriptions according to the extended BBCH scale. Grape and wine research **1**, 100-103.

Ge SX, Jung D, Yao R. 2020. ShinyGO: a graphical gene-set enrichment tool for animals and plants. Bioinformatics. **36**, 628-2629.
